# Diagnostic and therapeutic management of severe acute pancreatitis. Evidence based medicine (EBM) clinical practice guidelines

**DOI:** 10.20452/wiitm.2025.17941

**Published:** 2025-04-09

**Authors:** Jacek Szeliga, Mateusz Jagielski, Jacek Sobocki, Michał Borys, Mirosław Czuczwar, Adam Durczyński, Tomasz Gach, Piotr Hogendorf, Andrzej Kawiński, Katarzyna Kuśnierz, Piotr Major, Michał Mik, Michał Pędziwiatr, Paweł Piwowarczyk, Marek Sierżęga, Michał Spychalski, Wojciech Szczeklik, Mirosław Szura, Mateusz Wierdak, Witold Zgodziński, Andrzej Budzyński, Stanisław Hać, Marek Jackowski, Wojciech Kielan, Michał Kukla, Sławomir Mrowiec, Piotr Myśliwiec, Jerzy Sieńko, Maciej Słodkowski, Wiesław Tarnowski, Grzegorz Wallner, Marek Zawadzki, Krzysztof Zieniewicz

**Affiliations:** Department of General, Gastroenterological and Oncological Surgery, Collegium Medicum in Bydgoszcz, Nicolaus Copernicus University in Torun, Toruń, Poland; Department of General Surgery and Clinical Nutrition, Centre of Postgraduate Medical Education in Warsaw, Warszawa, Poland; Faculty of Medicine, Centre of Oncology, The John Paul II Catholic University of Lublin, Lublin, Poland; Department of Anesthesiology and Intensive Therapy, Multispecialist Regional Hospital in Gorzow Wielkopolski, Gorzów Wielkopolski, Poland; Department of General and Transplant Surgery, Norbert Barlicki Memorial University Teaching Hospital, Medical University of Lodz, Łódź, Poland; Department of Surgery, Institute of Physiotherapy, Faculty of Health Sciences, Jagiellonian University Medical College, Kraków, Poland; Department of General and Colorectal Surgery, The Military Medical Academy Memorial Teaching Hospital No. 2 in Lodz, Łodź, Poland; Department of Gastrointestinal Surgery, Medical University of Silesia, Katowice, Poland; Second Department of General Surgery, Jagiellonian University Medical College, Kraków, Poland; Department of General and Colorectal Surgery, Medical University of Lodz, Łódź, Poland; Faculty of Medicine, Institute of Medical Sciences, The John Paul II Catholic University of Lublin, Lublin, Poland; First Department of Surgery, Jagiellonian University Medical College, Kraków, Poland; Department of General and Oncological Surgery, Medical University of Lodz, Łódź, Poland; Specialist Hospital Brzeziny, Brzeziny, Poland; Center for Intensive Therapy and Perioperative Medicine, Jagiellonian University Medical College, Kraków, Poland; First Department of General Surgery and Transplant Surgery and Nutritional Treatment, Medical University of Lublin, Lublin, Poland; Department of General and Oncologic Surgery, Ludwik Rydygier Memorial Hospital in Krakow, Kraków, Poland; Department of Surgical Oncology, Transplant Surgery and General Surgery, Medical University of Gdansk, Gdańsk, Poland; University Centre of General and Oncological Surgery, Wroclaw Medical University, Wrocław, Poland, Poland; Department of Internal Medicine and Geriatrics, Jagiellonian University Medical College, Kraków, Poland; Endoscopy Department, University Hospital No. 1 in Krakow, Kraków, Poland; First Clinical Department of General and Endocrine Surgery, Medical University of Bialystok, Białystok, Poland; Institute of Physical Culture Sciences, University of Szczecin, Poland; National Consultant in General Surgery, Poland; Department of General, Gastroenterological and Oncological Surgery, Medical University of Warsaw, Warszawa, Poland; Department of General, Oncologic and Bariatric Surgery, Centre of Postgraduate Medical Education, Orłowski Hospital, Warszawa, Poland; Second Chair and Department of General, Gastrointestinal Surgery and Surgical Oncology of the Digestive Tract, Medical University of Lublin, Lublin, Poland; Faculty of Medicine, Wroclaw University of Science and Technology, Wrocław, Poland; Department of General, Transplant and Liver Surgery, Medical University of Warsaw, Warszawa, Poland

## BACKGROUND

The following guidelines were developed at the initiative of the Association of Polish Surgeons.

In preparing this paper, the authors conduct‑ ed a thorough analysis of the current literature on the management of acute surgical conditions.

A summary was drawn up following a comprehensive review of studies from the past sever‑ al years. The main object of the summary was to dissect current knowledge on the approaches to optimize the management of severe acute pancreatitis (AP).

The recommendations have been developed on the basis of a review of literature from the period of 1983–2024 as available from PubMed, Medline, and Cochrane Library databases, with a particular focus on systematic reviews and clinical recommendations published by recognized scientific societies in the period of 2020–2024. The query returned a total of 221 papers that were further subjected to an in‑depth analysis.

The following recommendations are general in nature and require case‑by‑case analysis and adaptation to specific clinical setting. Each recommendation was further graded according to the Grading of Recommendations Assessment, Development, and Evaluation (GRADE) criteria with the strength of the published scientific evidence being taken into account (TABLE 1 and Table 2).(Guyatt et al., 2008) In addition, the guidelines have received a recommendation from the National Consultant in General Surgery.

The recommendations will be updated every 5 years unless significant changes in the routine diagnostics and treatment of AP shall be made on the basis of strong scientific evidence, requiring immediate modification of the management guidelines.

The development of these recommendations had been planned and executed in the following stages:

1. Development of process and document plans, identification and invitation of experts;
2. Review of the literature and preliminary formulation of recommendations with annotations (all authors);
3. Formulation of a preliminary version of the guidelines;
4. Revision of the preliminary version and preparation of an assessment version;
5. Evaluation and revision using the Delphi method;
6. Formulation of the revised document (all authors);
7. Re‑evaluation and revision (invited experts);
8. If further revision was required: repetition of steps 6 and 7 (all authors);
9. Formulation of the final document (all authors).

## PROCESS FLOW

The recommendations were formulated in directive language and evaluated using the Delphi method. The preliminary document, consisting of annotated recommendations, was reviewed by an extended panel of experts using the following acceptance scale: 3 – strong acceptance; 2 – reserved acceptance; 1 – strongly reserved acceptance; 0 – rejection.

Numerous corrections and consensual agreements were made as early as at the document’s development stage, thus avoiding repeated iterations at the subsequent stages. The document was then evaluated by the entire team (second iteration). The recommendations with the aver‑ age acceptance score of >2 were defined as strong,

those with the average acceptance score of ≤2 and ≥1 were defined as weak, while the recommendations with the average acceptance score of <1 were rejected. All comments by the experts were incorporated into the recommendation text. Due to the highest strength of all recommenda‑ tions and the lack of suggestions for revisions, the Delphi process was terminated. A total of 32 experts—members of the Association of Polish Surgeons—participated in the process of formulating and evaluating the recommendations us‑ ing the Delphi method.

## RECOMMENDATIONS 

A summary of the recommendations, including the average acceptance scores, suggestions from the objecting experts, and the recommendation strengths are shown in TABLE 3. The wording “We recommend” is used to emphasize a consensus statement regarding the benefits derived by the patient from the procedure; the recommendation should be followed whenever possible. The wording “We propose” is used to suggest that the patient may benefit from the procedure; the procedure should be taken into account in the therapeutic decision‑making process. The wording “We do not recommend” is used to emphasize a consensus statement regarding the increased risk or a lack of additional ben‑ efits derived by the patient from the procedure.

### Abbreviations

ACS – abdominal compartment syndrome AP – acute pancreatitis

ARDS – acute respiratory distress syndrome BISAP – bedside index for severity in acute pancreatitis

BMI – body mass index CI – confidence interval CRP – C‑reactive protein

CT – computed tomography

CTSI – computed tomography severity index ERCP – endoscopic retrograde cholangiopancreatography

EUS – endoscopic ultrasonography IAH – intra‑abdominal hypertension IAP – intra‑abdominal pressure

ICU – intensive care unit

LMWH – low‑molecular‑weight heparin MD – mean difference

MODS – multiorgan dysfunction syndrome MR – magnetic resonance

MRCP – magnetic resonance cholangiopancreatography NNT – number needed to treat

NSAID – nonsteroidal anti‑inflammatory drugs OR – odds ratio

PEP – post‑ERCP pancreatitis

PK/PD – pharmacokinetic / pharmacodynamic profile

PPC – pancreatic pseudocyst PPI – proton pump inhibitor

RCT – randomized controlled trial RR – relative risk

RR – relative risk

SIRS – systemic inflammatory response syndrome

TG – triglycerides US – ultrasound

VARD – video‑assisted retroperitoneal debridement

VTE – venous thromboembolism

## 1. INTRODUCTION

Acute pancreatitis (AP) is an inflammation of the pancreas and the surrounding tissues, one of the most common of all acute gastrointestinal conditions requiring hospitalization. AP is characterized by an increasing incidence, extremely variable, marked, and unpredictable dynamics, significant risk of complications responsible for the unfavorable course of the disease, and high mortality. To this day, it remains unclear whether the infection of pancreatic necrosis or rather the concomitant organ failure is the decisive prognostic factor.(Hu et al., 2023) All of this makes predicting the course of AP and optimizing therapy a formidable challenge, even for the most experienced clinicians. To date, targeted therapy has not been developed, the best management consisting in an individualized approach based on certain general principles derived from cur‑ rent knowledge of the complex pathology of AP. Thus, the following recommendations are based on the most recent, reliable scientific data on the diagnosis and treatment of AP, especially its severe clinical manifestation, their main objective being to provide comprehensive support for diagnostic and therapeutic decision‑making.

## 2. EPIDEMIOLOGY

The worldwide incidence of AP ranges from 10 to 90 cases /100000 population / year (average of about 34/100000/year). The estimated incidence in Poland, amounting to 80 cases/100 000 adults/year, is therefore among the highest in the world; the incidence is statistically higher by about 10%–30% for the male population, as compared with the female population. Also, the overall mortality rate is relatively very high, oscillating between 5% and 10%, largely depending on the clinical form of AP.

As shown in a recently published literature review analyzing the long‑term global epidemiologic trends, the overall number of new diagnoses of AP in the Western world is increasing by about 3% each year.(Iannuzzi et al., 2022) Unfortunately, the prevalence of this pathology in the Polish population is also steadily increasing. Realistic estimates suggest that the severe form of the disease, being associated with a markedly worse prognosis, typically accounts for 10%–20% of all cases of diagnosed AP, and is beyond doubt also on an upward trend.

**TABLE 1:** Grading of Recommendations Assessment, Development and Evaluation (GRADE), based on Guyatt et al(Guyatt et al., 2008)^,^(Leppäniemi et al., 2019)

Grade of recommendation	Clarity of risk / benefit	Quality of supporting evidence	Implications
1A			
Strong recommendation, high‑quality evidence	Benefits clearly outweigh the risks and burdens, or vice versa	RCTs without important limitations or overwhelming evidence from observational studies	Strong recommendation, applies to most patients in most circumstances without reservation
1B			
Strong recommendation, moderate‑quality evidence	Benefits clearly outweigh the risks and burdens, or vice versa	RCTs with important limitations (inconsistent results, methodological flaws, indirect analyses, or imprecise conclusions) or exceptionally strong evidence from observational studies	Strong recommendation, applies to most patients in most circumstances without reservation
1C			
Strong recommendation, low‑quality or very low‑quality evidence	Benefits clearly outweigh the risks and burdens, or vice versa	Observational studies or case series	Strong recommendation but subject to change when higher‑quality evidence becomes available
2A			
Weak recommendation, high‑quality evidence	Benefits closely balanced with the risks and burdens	RCTs without important limitations or overwhelming evidence from observational studies	Weak recommendation, best action may differ depending on the patient, treatment circumstances, or social values
2B			
Weak recommendation, moderate‑quality evidence	Benefits closely balanced with the risks and burden	RCTs with important limitations (inconsistent results, methodological flaws, indirect, or imprecise) or exceptionally strong evidence from observational studies	Weak recommendation, best action may differ depending on the patient, treatment circumstances, or social values
2C			
Weak recommendation, low‑quality or very low‑quality evidence	Uncertainty in the estimates of benefits, risks, and burdens; benefits, risk, and burdens may be closely balanced	Observational studies or case series	Very weak recommendation; alternative treatments may be equally reasonable and merit consideration

Abbreviations: RCT, randomized controlled trial

**Table 2:** Criteria for assessing data quality(Guyatt et al., 2008)

Quality of data	Description
High	There is ≥1 high‑quality, well‑conducted randomized controlled trial that provides consistent results and allows for direct application of conclusions. Further research is highly unlikely to significantly affect the estimated effect.
Moderate	There are randomized studies available, but they have significant limitations, such as biased evaluation of treatment effects, a large number of patients lost to follow‑up, lack of blinding, unexplained heterogeneity, indirect inferences related to similar (but not identical) study populations, very small patient groups, or consideration of a limited number of events (end points). Additionally, there are data from well‑designed controlled studies without randomization, well‑prepared cohort, or case‑control analytic studies, and numerous case series with or without intervention. Further research is likely to have an important impact on the estimated effect and may change it.
Low	Observational studies, typically of low quality due to the risk of bias. Further research will almost certainly have a significant impact on the estimated effect and will most likely alter it.
Very Low	The data are conflicting, of low quality, or lack results, making it impossible to determine the balance of benefits and risks. Any estimated effect is highly uncertain as evidence or is even unavailable, making it impossible to draw conclusions.

The mortality rate for this most severe necrotizing form of the disease complicated by an infection can be as high as 50%.

## 3. ETIOLOGY, PATHOMECHANISM, DIAGNOSIS

AP can be triggered by a variety of factors, such as:

- cholelithiasis;
- alcohol consumption;
- iatrogenic causes (procedures within the bil‑ iopancreatic field, including endoscopic retro‑ grade cholangiopancreatography [ERCP] inducing the so‑called acute post‑ERCP pancreatitis [PEP], pancreatic resections);
- medications (6‑mercaptopurine, azathioprine, isoniazid, loop diuretics, valproic acid, estrogens, tamoxifen, sulfonamides, tetracyclines, glucagon‑like peptide‑1 analogs, corticosteroids, octreotide, 5‑aminosalicylic acid, methyldopa);
- metabolic disorders: hypertriglyceridemia (especially hyperlipidemia types I, IV, and V), hypercalcemia (including hyperparathyroidism, chronic renal failure);
- tumors of the biliopancreatic field (solid tumors of the pancreas, cystic tumors of the pancreas, tumors of the greater duodenal papilla);
- abdominal injuries;
- autoimmune processes (vasculitis, systemic lu‑ pus erythematosus, Sjögren syndrome);
- anatomical variants (bicuspid pancreas, peri‑ ampullary diverticulum);
- genetic disorders (hereditary pancreatitis, cys‑ tic fibrosis, α1‑antitrypsin deficiency);
- viral infections (Coxsackie virus, cytomegalo‑ virus, Epstein–Barr virus, HIV, mumps virus, rubella virus, chickenpox virus);
- parasitic infestations (Ascaris lumbricoides, Cryptosporidium, Clonorchis sinensis, Microsporidia);
- bacterial infections (Campylobacter jejuni, Legionella spp., Leptospira spp., Mycobacterium avium, M. tuberculosis, Mycoplasma spp.)
- idiopathic causes.

More recently, selected complications of obesity have been emphasized as independent risk factors for the development of AP. These primarily include hyperlipidemia, cholelithiasis, or diabetes.(Konończuk et al., 2018)^,^ (Kozieł & Głuszek, 2016)^,^ (Qin et al., 2024) The pathophysiology of AP has not been thoroughly explained to date. The natural history of AP involves an imbalance in mechanisms that inhibit and stabilize enzyme activity in pancreatic cells to promote premature activation of enzymes within the organ itself. This leads to auto‑digestion of the pancreas and, as the disease progresses, digestion of the surrounding tissues and neighboring organs. This process then becomes the impetus for the development of systemic inflammatory response syndrome (SIRS) and immune responses involving numerous mediators and specific cells that play the key role in the severe forms of AP and distant organ damage.(Konończuk et al., 2018)^,^(Giakoumakis et al., 2022)

In every case of AP diagnosis, it is important that the etiology of the disease is being sought for from the outset, as it translates into therapeutic decisions. In Poland, in the adult population, the most common (about 80% of cases) causes of AP include gallstones and alcohol‑related back‑ ground. In the pediatric population, on the other hand, blunt abdominal trauma (bicycle handlebar injuries) and mumps virus infections are the pre‑ dominant etiological factors.

The diagnosis of idiopathic AP should not be misused as it frequently masks undiagnosed causes of the disease, such as lithogenic bile, microlithiasis, or pancreatic neoplasm (including cystic neoplasms). According to the available literature data, idiopathic AP is eventually diag nosed in about 10% of patients.(Heckler et al., 2021)^,^(Strzelec et al., 2020)^,^(G et al., 2019)

### 3.1 We recommend that the diagnosis of AP be based on meeting of the following 3 criteria: 1) characteristic epigastric pain of typical location; 2) serum pancreatic enzyme activity 3 times the upper limit of normal values; 3) typical imaging findings. (GRADE 1C)

If the first 2 of the aforementioned criteria (related to the clinical presentation and laboratory blood test results) are met, pancreatic imaging is not necessary to confirm the diagnosis of AP. However, it should be remembered that for the correct diagnosis of the disease—especially in the cases of diagnostic doubts—differential diagnosis is very important. In such cases, it is reasonable to extend the diagnostics to include additional imaging studies so as to assess the local condition of the pancreas.

Having taken the complaints suggestive of AP into account in the diagnostic process, it must be borne in mind that despite the high sensitivity of serum amylase and lipase activity determinations, concentrations of these enzymes may also be increased in other pathological conditions, some of them presenting with a clinical course similar to that of AP. Therefore, the differential diagnosis should include:

- perforation of the gastrointestinal tract in the course of gastric or duodenal ulcer disease;
- acute appendicitis;
- acute intestinal ischemia;
- dissecting aneurysm of the aorta;
- ectopic pregnancy;
- inferior wall myocardial infarction;
- kidney damage;
- liver damage;
- choledocholithiasis;
- metabolic acidosis;
- tumors (ovarian cancer, pancreatic cancer; myeloma, pheochromocytoma);
- inflammatory bowel diseases.(Banks et al., 2013)^,^(Thoeni, 2012)^,^(Boxhoorn et al., 2020)^,^(Tenner et al., 2013)^,^(Mederos et al., 2021)^,^(Gardner, 2021)^,^(Forsmark et al., 2016)^,^(Greenberg et al., 2016)^,^(Lee & Papachristou, 2019)^,^(Lam & Hyperamylasemia, n.d.)

**TABLE 3:** A summary of the recommendations along with a comprehensive evaluation, including: expert assessment, data quality criteria, and evaluation according to the GRADE system(Guyatt et al., 2008) (continued from the previous page)

Recommendation	Dolphi rating/strenght of recommendation*	Data quality	Grade of recommendation
Diagnosis			
3.1. We recommend that the diagnosis of AP be based on meeting 2 of the following 3 criteria: – characteristic epigastric pain of typical location, – serum pancreatic enzyme activity 3 times the upper limit of normal values, – typical imaging findings.	3.0/Strong	Moderate	1C
3.2. If the most common causes of AP are ruled out, we recommend paying special attention to the potential etiology related to proliferative diseases (tumors of the pancreas, duodenum, major duodenal papilla).	2.9/Strong	Moderate	1C
3.3. After ruling out cholelithiasis, tumor of the biliopancreatic field, or alcoholic etiology of AP, we recommend that blood TG and calcium levels be determined and potential disease entities leading to increased blood calcium levels (e.g. chronic renal failure, hyperparathyroidism) be ruled out. TG levels of >1000 mg/dl are generally indicative of the etiology of the disease.	2.7/Strong	Low	2C
3.4. Prior to establishing a diagnosis of idiopathic AP, we recommend performing MRCP or EUS to detect possible underlying causes.	2.8/Strong	Moderate	1C
Prediction, stratification, and ongoing severity assessment			
4.1. We recommend the use of prognostic scales to assess the predicted clinical course of AP.	2.7/Strong	Moderate	1C
4.2. We recommend the use of the BISAP prognostic scale to assess the risk of severe AP.	2.6/Strong	Moderate	1C
The role of imaging studies (diagnosis/monitoring)			
5.1. We recommend that a routine US scan of the abdomen be performed on every AP patient upon their admission to the hospital.	2.8/Strong	Moderate	1C
5.2. We do not recommend CT scans of the abdomen being routinely acquired for every AP patient upon hospital admission.	2.2/Strong	Moderate	1C
5.3. If ductal microcalcification is suspected and visible on classic US, qualification for EUS or MRCP should be considered.	2.8/Strong	Moderate	1B
5.4. We recommend that contrast-enhanced CT scans be performed in patients with severe clinical forms of AP ≥72 hours after the onset of symptoms.	2.8/Strong	Low	1C
5.5. In patients sensitized to iodine contrast, individuals with renal failure, and pregnant women, we propose that MRI be performed instead of CT.	2.7/Strong	Moderate	1B
5.6. We recommend that contrast-enhanced CT scans be performed as part of disease monitoring, in the event of any worsening of the patient’s clinical condition or lack of expected improvement, or if surgical intervention is being planned.	2.9/Strong	Moderate	1B
Classifications			
6.1. We propose the 2012 revision of the Atlanta Classification be used to standardize the nomenclature and definitions of AP.	2.9/Strong	Moderate	1C
Prevention			
7.1. We recommend perioperative use of diclofenac administered rectally at a dose of 100 mg as a preventive measure in patients at a high risk of PEP.	2.6/Strong	High	1A
Conservative treatment: targeted therapy			
8.1. We do not recommend the routine use of somatostatin, its analogues, protease inhibitors, antioxidants, glucagon, or insulin in the treatment of AP patients.	2.8/Strong	High	1A
8.2. We propose that AP patients with features of IAH be treated with neostigmin.	1.9/Weak	Moderate	2B
8.3. We recommend that PPIs be used only as an adjunct to standard AP treatment if significant comorbidities listed as indications for which therapy are present, or if an increased risk of gastrointestinal bleeding or PPC is recognized.	2.7/Strong	Moderate	2A
Intensive care			
9.1. We recommend that patients with severe AP and concomitant MODS or at a risk of MODS be treated in the ICU setting. The final classification for ICU treatment shall be based on the current guidelines of the Society of Anesthesiology and Intensive Therapy.	2.9/Strong	Low	1C
9.2. In patients with organ failure, we recommend that basic vital signs be monitored continuously.	2.9/Strong	Low	1C
9.3. In patients admitted to the ICU for severe AP, we recommend determination of the plasma levels of alanine aminotransferase, aspartate aminotransferase, bilirubin, TG, calcium, phosphorus, creatinine, and urea as well as arterial blood gas levels.	2.7/Strong	Moderate	1C
9.4. In order to assess the presence of infected pancreatic necrosis in patients admitted to the ICU for severe AP, we recommend that CRP and procalcitonin levels be determined and an abdominal CT scan be performed.	2.9/Strong	Moderate	1B
9.5. We recommend IAP monitoring in severe AP patients treated in the ICU setting, particularly in the mechanically ventilated patients.	2.8/Strong	Moderate	1B
9.6. We do not recommend intensive fluid therapy due to the increased incidence of respiratory failure and IAH.	2.5/Strong	Moderate	1B
9.7. We recommend goal-directed fluid therapy guided by dynamic indices and arterial blood lactate levels.	2.8/Strong	Moderate	1B
9.8. We suggest using a vasoconstrictor drug (norepinephrine) for arterial hypertension that persists despite adequate fluid therapy.	2.6/Strong	Moderate	1B
9.9. We recommend fluid therapy based on balanced crystalloid solutions (eg, Ringer lactate).	2.8/Strong	Moderate	1B
9.10. We do not recommend using 0.9% NaCl solution or synthetic colloids (hydroxylated starch, gelatins, dextrans).	2.4/Strong	Moderate	1B
9.11. In patients diagnosed with AP, we recommend multimodal pain therapy based on nonopioid and opioid analgesics. We do not recommend intravenous infusions of local anesthetic agents as a means of pain management.	2.7/Strong	Moderate	1B
9.12. In critically ill patients, we recommend that thoracic epidural anesthesia be used as an alternative pain treatment.	2.4/Strong	Moderate	1B
Intra-abdominal hypertension			
10.1. We recommend IAP be measured and IAH treatment be implemented whenever it is suspected.	2.7/Strong	Moderate	1C
10.2. Once IAH is diagnosed, we first recommend placing a patient in the supine position, their legs slightly bent, followed by the delivery of appropriate analgesia and sedation, and decompression of the stomach (using a nasogastric tube), bladder (using a catheter), and colon (using a drain inserted into the rectum).	2.6/Strong	Moderate	1C
10.3. We recommend paracentesis/transcutaneous drainage in the cases of IAH-complicated AP accompanied by ascites.	2.4/Strong	Low	2C
10.4. In the absence of the therapeutic effect of nonsurgical procedures used in the treatment of IAH/ACS, we recommend that minimally invasive techniques and procedures be used; open abdominal treatment should follow if the former are ineffective.	2.4/Strong	Low	2C
Antibiotic prevention and treatment			
11.1. We do not recommend prophylaxis with antimicrobial drugs, regardless of the severity of AP.	2.6/Strong	High	1A
11.2. We do not recommend the use of pro- or symbiotics, regardless of the severity of AP.	2.5/Strong	Moderate	1B
11.3. We do not recommend the use of antimicrobial therapy to treat infected pancreatic necrosis on the basis of clinical symptoms and CRP measurements alone. Diagnosis of the infection of pancreatic necrosis should be based on imaging studies (eg, contrast-enhanced abdominal CT scans). However, we recommend that procalcitonin levels be monitored to optimize the use of antimicrobial drugs.	2.7/Strong	High	1A
11.4. We recommend that the treatment of infected necrotic foci be started with antimicrobial drugs rather than a surgical intervention (step 0).	2.4/Strong	Moderate	1C
11.5. If the infection of pancreatic necrosis is confirmed, we recommend that empirical antimicrobial therapy targeting the Enterobacteriaceae, Enterococcus faecium, and Pseudomonas aeruginosa strains be implemented. While empirical treatment should be modified according to the results of microbial cultures, obtaining the material for microbiological examinations is not necessary for the initiation of treatment.	2.8/Strong	Moderate	1C
11.6. It is not possible to recommend specific antimicrobial drugs for use in the empirical treatment of infected pancreatic necrosis. The choice should be based on local infection maps, drug resistance prevalence, and the PK/PD profile of the antimicrobial drug.	2.6/Strong	Very low	2C
11.7. We recommend that antimicrobial drugs be dosed according to their PK/PD profiles.	2.9/Strong	Moderate	1C
11.8. We recommend that empirical antifungal therapy be followed only in patients at a high risk of invasive fungal infection or those with confirmed fungal infection.	2.6/Strong	Moderate	1B
Thromboprophylaxis/anticoagulation			
12.1. In all patients hospitalized for AP, we recommend that the risk of VTE be assessed using dedicated scales (eg, the Padua scale for conservatively treated patients, the Caprini scale for surgically treated patients). In the cases of moderate-to-high risk of VTE, we recommend that thromboprophylaxis be implemented according to current guidelines.	2.8/Strong	Moderate	1B
12.2. We propose that prophylactic LMWH doses be used in all cases of moderate-to-severe forms of AP, provided the absence of specific contraindications or increased risks associated with such treatment.	2.8/Strong	Moderate	1B
Nutrition/nutritional treatment			
13.1. We recommend that nutrition in an amount tailored to the metabolic capabilities of the AP patient be implemented as soon as the prognostic factors of a severe course are established, cell perfusion is ensured, and severe water-electrolyte and acid-base disorders are controlled.	3.0/Strong	Moderate	1B
13.2. We recommend gastrointestinal feeding as the preferred method for the delivery of nutrients.	2.8/Strong	High	1A
13.3. When gastric motor function is preserved, we propose that intragastric nutrition be used, the enteral nutrition—administered via the ligament of Treitz—being recommended for patients with gastric stasis.	2.9/Strong	Moderate	1B
13.4. We recommend that supplemental or total parenteral nutrition be used in patients with limited or total gastrointestinal failure or IAH preventing the delivery of full/minimal enteral nutrition.	2.9/Strong	Moderate	1B
13.5. We recommend that the individual components of the feeding mixture be selected according to metabolic limits.	2.9/Strong	Low	1C
Diagnosis of infected pancreatic necrosis			
14.1. We do not recommend routinely performing fine-needle aspiration biopsy to confirm infection, as it frequently leads to secondary infection of the necrotic tissue.	2.8/Strong	Moderate	1C
14.2. For the diagnosis of infected pancreatic necrosis, we recommend reliance on indirect evidence, that is, the presence of gas bubbles outside the gastrointestinal lumen on multiphase abdominal and pelvic CT scans, after exclusion of gastrointestinal perforation.	2.7/Strong	Moderate	1C
Diagnosis and classification of AP sequelae			
15.1. We propose that the 2012 revision of the Atlanta Classification be used to describe the complications and sequelae of AP.	2.9/Strong	Moderate	1C
Differences in the management strategy and practice depending on the etiology of AP			
16.1. In the case of biliary AP with cholangitis, we recommend that ERCP with endoscopic sphincterotomy and evacuation of deposits be performed within 24 hours.	2.8/Strong	High	1A
16.2. In the case of biliary AP with persistent biliary obstruction and no cholangitis, we recommend that ERCP with endoscopic sphincterotomy and evacuation of deposits be performed within 72 hours.	2.9/Strong	High	1A
16.3. We do not recommend performing ERCP to treat biliary AP in patients with cholecystolithiasis presenting with no features of persistent biliary obstruction or cholangitis.	2.5/Strong	High	1A
16.4. Cholecystectomy is indicated in the treatment of AP in patients with confirmed cholecystolithiasis. Perioperative risks in acute pancreatitis as well as risks of complications of cholelithiasis should be taken into account when deciding on the timing of the procedure. We propose that cholecystectomy be performed in patients with mild AP against the background of cholecystolithiasis during the same hospital stay.	2.4/Strong	High	1A
16.5. In the case of gallstone-related AP involving the development of peripancreatic fluid reservoirs, we recommend that cholecystectomy be postponed until resorption/stabilization of the reservoir and resolution of inflammation.	2.7/Strong	Moderate	1B
16.6. For significant hypertriglyceridemia, we suggest insulin infusion, especially in diabetic patients, and for persistently high TG values in patients with severe (>11.3 mmol/l) or very severe (>22.4 mmol/l) hypertriglyceridemia, we recommend considering therapeutic plasma exchange, especially if there is no response to conservative treatment (fibrates, heparin, insulin).	2.4/Strong	Very low	2C
Qualification for surgical treatment: intervention timing and purpose			
17.1. On the basis of the available data, no recommendations can be made regarding the timing of surgical intervention in severe forms of AP, regardless of the type of such intervention (open, minimally invasive, or endoscopic).	2.5/Strong	Moderate	2B
Step-up approach			
18.1. We suggest that the interventional treatment of local complications of AP be started with percutaneous or endoscopic drainage techniques followed by subsequent re-evaluation of indications for further, more invasive surgical treatment.	2.8/Strong	Moderate	1B
18.2. We propose that minimally invasive techniques be used in clinical cases requiring the removal of infected necrotic tissue.	2.7/Strong	Moderate	1B
Step-up: endoscopic treatment of complications			
19.1. When qualifying patients for endoscopic treatment of AP sequelae in the form of postinflammatory pancreatic and peripancreatic fluid reservoirs, we recommend that establishing an appropriate drainage system be the cornerstone of therapy.	2.8/Strong	Moderate	2B
19.2. We recommend that the choice of drainage method be dependent on the location and type of postinflammatory pancreatic or peripancreatic fluid reservoirs.	2.7/Strong	Moderate	2B
19.3. We propose endoscopic transmural drainage (through the wall of the upper gastrointestinal tract, transgastric or transduodenal) under EUS guidance as the preferred endointerventionist approach.	2.8/Strong	Moderate	2B
19.4. We recommend that active drainage through a nasal drain be used in the case of fluid reservoirs in the course of necrotizing AP.	2.3/Strong	Moderate	2B
19.5. We propose that a step-up approach be followed in the case of fluid reservoirs in the course of necrotizing AP, including potential increase in the number of the points of access to the necrotic tissue or intensification of treatment by means of direct endoscopic necrosectomy.	2.7/Strong	Moderate	2B
Step-up approach: surgical treatment of complications			
20.1. In clinical cases requiring removal of infected necrotic tissue, we suggest that the minimally invasive technique in which the particular institution has the greatest experience be the method of choice.	2.8/Strong	Moderate	1B
20.2. We do not recommend routine use of open abdominal surgery in patients with AP without concomitant ACS.	2.6/Strong	Very low	2C
20.3. We recommend that negative pressure dressings be used in patients treated with open abdominal surgery.	2.2/Strong	Very low	2C
Criteria for completion of inpatient treatment			
21.1. We propose that the inpatient treatment of patients with severe AP be completed in accordance with the Hungarian Pancreatic Study Group criteria after the completion of invasive treatment and antibiotic therapy and with patient’s overall condition being taken into account.	2.4/Strong	Very low	2C
Prognosis and outpatient surveillance			
22.1. We recommend that patients be followed for up to 2 years after the completion of AP treatment.	2.3/Strong	Moderate	1C
22.2. We recommend that follow-up abdominal US be performed at 3 and 6 months after hospital discharge.	2.4/Strong	Moderate	1C
22.3. In the cases of BISAP score of ≥2 and CTSI (Balthazar) score of ≥3, we recommend that contrast-enhanced CT scan be acquired at 1–3 months after hospital discharge.	2.6/Strong	Moderate	1C
22.4. Due to the increased risk of pancreatic cancer, we suggest that CT scans be repeated after 1 and 2 years.	2.4/Strong	Moderate	1C
22.5. If exocrine pancreatic insufficiency is suspected, we recommend that the activity of FE-1 be assessed by means of an FE-1 assay or, alternatively, a ¹³C-labeled mixed triglyceride breath test.	2.5/Strong	Moderate	1C
22.6. We recommend monitoring of endocrine pancreatic insufficiency as manifested by prediabetes or diabetes (type 3c) for 1–2 years after the episode of AP on the basis of fasting blood glucose, HbA_₁c_, and glucose load test.	2.6/Strong	Moderate	1C

a The average Delphi voting result scale can be described as follows: 3 – strong acceptance, 2 – reserved acceptance, 1 – strongly reserved acceptance, 0 – rejection.

Abbreviations: ACS, abdominal compartment syndrome; AP, acute pancreatitis; ARDS, acute respiratory distress syndrome; BISAP, bedside index for severity in acute pancreatitis; BMI, body mass index; CRP, C‑reactive protein; CT, computed tomography; CTSI, computed tomography severity index; ERCP, endoscopic retrograde cholangiopancreatography; EUS, endoscopic ultrasonography; FE‑1, fecal elastase 1; HbA1c, glycated hemoglobin; IAH, intra‑abdominal hypertension; IAP, intra‑abdominal pressure; ICU, intensive care unit; LMWH, low‑molecular‑weight heparin; MD, mean difference;

MODS, multiorgan dysfunction syndrome; MRI, magnetic resonance imaging; MRCP, magnetic resonance cholangiopancreatography; NNT, number needed to treat; NSAID, nonsteroidal anti‑inflammatory drugs; OR, odds ratio; PEP; post‑ERCP pancreatitis; PK/PD, pharmacokinetic / pharmacodynamic profile; PPC, pancreatic pseudocyst; PPI, proton pump inhibitor; RR, relative risk; SIRS, systemic inflammatory response syndrome; TG, triglycerides; US, ultrasound; VARD, video‑assisted retroperitoneal debridement; VTE, venous thromboembolism; others, see TABLE 1

### 3.2. If the most common causes of AP are ruled out, we recommend paying special attention to the potential etiology related to proliferative diseases (tumors of the pancreas, duodenum, major duodenal papilla). (GRADE 1C) 

In an epidemiological study involving large populations of Danish and American patients diagnosed with pancreatic malignancies, Kirkegård et al(Kirkegård et al., 2020)^,^(Kirkegård et al., 2020) found the comorbid AP symptoms in 1%–1.4% and 5.9% of participants, respectively.

### 3.3. After ruling out cholelithiasis, tumor of the biliopancreatic field, or alcohol‑related etiology of AP, we recommend that blood triglyceride (TG) and calcium levels be determined and potential disease entities leading to increased blood calcium levels (eg, chronic renal failure, hyperparathyroidism) be ruled out. TG levels of >1000 mg/dl are generally indicative of the etiology of the disease. (GRADE 2C)

When determining the causes of AP, it is important to keep in mind the increasing number of cases of solid and cystic tumors of the pancreas and major duodenal papilla which are the direct cause of the disease. A similar upward trend applies to iatrogenic AP due to a significant prevalence of ERCP and interventional radiology pro‑ cedures (most frequently developing in an isch‑ emic mechanism after embolization of vascular malformations within the pancreaticoduodenal field). Drug‑induced AP is rare (the literature contains descriptions of >120 different active substances used prior to its development). The disease can also occur after pancreatic sur‑ gery (known as stump pancreatitis), especially after pancreatoduodenectomy. In the case of in‑ creased serum calcium levels, all possible disease entities leading to hypercalcemia (chronic renal failure, hyperparathyroidism) should be taken into account; in the cases of hypertriglyceride‑ mia, the same applies to hyperlipidemia types I, IV, and V.(Lamassab et al., 2022)^,^(Piñerúa‐Gonsálvez et al., 2023)^,^(Qiu et al., 2023)^,^(Mignini et al., 2021)^,^(Lamassab et al., 2022)^,^(Otsuka et al., 2018)

### 3.4. Prior to establishing a diagnosis of idiopathic AP, we recommend performing magnetic resonance cholangiopancreatography (MRCP) or endoscopic ultrasound (EUS) to detect possible underlying causes. (GRADE 1C)

In a recent review, Nikpanah and Morgan(Nikpanah & Morgan, 2024) emphasize the importance of magnetic resonance imaging (MRI) in the diagnosis of nonstandard causes of AP, particularly due to the advantage of this modality in terms of image resolution or the ability to acquire functional data following the use of appropriate contrast techniques.

## 4. PREDICTION, STRATIFICATION AND ONGOING SEVERITY ASSESSMENT

### 4.2. We recommend the use of prognostic scales to assess the predicted clinical course of AP. (GRADE 1C)

The clinical course of AP is difficult to predict, especially in the early stages. Therefore, it is extremely important to determine the risk of developing the severe form of the disease. The clinical characteristics presented upon admission and indicative of this type of risk include:

- age of >55 years;
- obesity (body mass index [BMI] >30 kg/m2);
- accompanying disturbances of consciousness;
- comorbidities (eg, obesity, hypertriglyceridemia);
- SIRS;
- abnormalities in laboratory blood tests (increased urea, creatinine, and C‑reactive protein [CRP] levels or increased hematocrit value);
- abnormalities on imaging studies (pleural ef‑ fusions, infiltrative lesions within the lungs, extrapulmonary fluid reservoirs).

The aforementioned clinical presentations are components of prognostic scales that may be useful in determining the prognosis in AP patients.(Mederos et al., 2021)^,^(Gardner, 2021)^,^(Forsmark et al., 2016)^,^(Greenberg et al., 2016)^,^(Valverde‐Lopez et al., 2017)^,^(Papachristou et al., 2010)^,^(Gómez et al., 2010)^,^(Forsmark et al., 2016)

Some of the prognostic scales most commonly used in AP patients include:

- the Bedside Index for Severity in Acute Pancreatitis (BISAP); and
- the Ranson scale.

Less common prognostic scales include:

- the Harmless Acute Pancreatitis Score (HAPS);
- the Japanese Severity Score (JSS); and
- the Pancreatitis Outcome Prediction (POP).

### 4.2. We recommend the use of the BISAP prognostic scale to assess the risk of severe AP. (GRADE 1C) 

The BISAP scale is a widely used and simple scale assessing 5 clinical parameters during the first 24 hours of AP symptoms: blood urea nitrogen levels of >25 mg/dl (8.93 mmol/l), impaired consciousness, features of SIRS, age of >60 years, and pleural effusion. Each of the aforementioned criteria is assigned a score of 1. A score of ≥3 points is indicative of severe AP as early as on the first day of illness, with sensitivity and specificity of the cutoff value amounting to 75% and 98.9%, respectively. The BISAP can also be used on subsequent days as a prognos‑ tic scale to predict a severe clinical course or fa‑ tal outcome.(Greenberg et al., 2016)^,^(Valverde‐Lopez et al., 2017)^,^(Papachristou et al., 2010)

## 5.THE ROLE OF IMAGING STUDIES (DIAGNOSIS/MONITORING)

### 5.1. We recommend that a routine ultrasound (US) scan of the abdomen be performed in every AP patient upon their admission to the hospital. (GRADE 1C)

Performing an abdominal US on admission to the hospital facilitates the determination of the lithiatic etiology of AP and the planning of appropriate interventional (surgical or endoscopic) treatment. Sensitivity and specificity of US in the diagnosis of cholecystolithiasis exceed 90%; however, in the case of microcalcification (deposits of <3 mm in diameter), these parameters are reduced to about 65%. In some patients, US also facilitates visualization of radiological features of the pancreas: parenchymal edema, obliteration of the surrounding fatty tissue, or peripancreatic reservoirs. Evaluation of pleural cavities should also be a part of the examination in the cases of suspected AP. Indeed, pleural effusion as diagnosed on admission is an unfavorable prognostic factor for patients with AP. It should always be borne in mind that an unremarkable US image of the pancreas as acquired upon hospital admission does not exclude the diagnosis of AP, and that the radiological evaluation itself may be significantly hampered by lipedema or accumulation of intestinal gas.(Walkowska et al., 2022)^,^(Sirli & Sporea, 2010)

### 5.2. We do not recommend computed tomography (CT) scans of the abdomen being routinely acquired for every AP patient upon hospital admission. (GRADE 1C)

Abdominal CT scans as performed at the time of the patient’s admission have lower sensitivity and specificity in diagnosing cholelithiasis than abdominal US scans. This is due to low sensitivity of CT in detecting noncalcified and poorly calcified deposits. In a 2021 study including 174 AP patients having been subjected to CT examinations upon hospital admission, only 39% were found to present with radiographic features of pancreatitis. In the group of AP patients in whom both examinations (ie, CT and US scans) were performed, cholelithiasis was diagnosed in 37% of cases, whereas in those undergoing CT alone, this rate was 23%.(Lohse et al., 2021)

### 5.3. If ductal microcalcification is suspected and visible on classic US, qualification for EUS or MRCP should be considered. (GRADE 1B)

 In a meta‑analysis of 18 studies involving a total of 2366 patients, sensitivity and specificity of MRCP and EUS in diagnosing cholelithiasis were at the same high level of more than 90%. No statistical differences in sensitivity or specificity were found between the 2 modalities. The choice of modality should therefore be dictated by its availability or the presence of specific contraindications.(Giljaca et al., 2015)

### 5.4. We recommend that contrast‑enhanced CT scans be performed in patients with severe clinical forms of AP ≥72 hours after the onset of symptoms. (GRADE 1C)

An early CT scan may underestimate the area of necrosis within the pancreas and peripancreatic space, as full impairment of the organ’s perfusion and its consequences become apparent after about 7 days. Thus, in the first few days, the image of the pancreatic parenchyma visible on contrast‑enhanced CT may be irregular, blurred, and heterogeneous, with spatial restriction becoming apparent only at a later time. In such cases, repeating the examination after 5–6 days offers a chance to more accurately determine the ana‑ tomical extent of pancreatic necrosis.(Brizi et al., 2021)

### 5.5. In patients sensitized to iodine contrast, individuals with renal failure, and pregnant women, we propose that MRI be performed instead of CT. (GRADE 1B)

A review article published in 2024 highlighted the advantages of MRI in determining the etiology of AP, including the modality being used as a first‑line technique in patients sensitized to radiological contrast used in CT.(Nikpanah & Morgan, 2024) Also, according to the latest guidelines, the administration of an MRI contrast agent does not have to depend on renal function parameters.(Chmielewski et al., 2024)

### 5.6. We recommend that contrast‑enhanced CT scans be performed as part of disease monitoring, in the event of any worsening of the patient’s clinical condition or lack of expected improvement, or if surgical intervention is being planned. (GRADE 1C)

No scientific data are available to unequivocally suggest any specific regimen for radiological monitoring of severe AP. In the case of concomitant renal failure with estimated glomerular filtration rate of <30 ml/min/1.73 m2, MRI should be considered instead of CT in selected cases.(Chmielewski et al., 2024)

## 6. CLASSIFICATIONS

### 6.1. We propose the 2012 revision of the Atlanta Classification be used to standardize the nomenclature and definitions of AP. (GRADE 1C)

The current international interdisciplinary con‑ sensus document on the classification of the clinical and pathomorphological forms of AP and its sequelae is the 2012 revision of the Atlanta Classification. According to this document, AP is classified as mild, moderate, or severe, depending on the co‑occurrence and duration of organ failure which, in turn, is determined using a modified Marshall scale.

The pathomorphological classification of AP distinguishes between interstitial edematous and necrotic forms, which are defined on the basis of radiological assessment of the local progression of the disease, usually by means of a multi‑ phasic abdominal CT scan with intravenous contrast enhancement. Typically, the mild‑to‑moderate clinical forms of AP correspond to the interstitial and edematous forms, while the necrotic form is usually associated with a severe clinical course. The degree of morphological abnormalities developing in the course of AP can be accurately determined using a scale based on the CT Severity Index (CTSI) developed by Balthazar et al.(Banks et al., 2013)^,^(Thoeni, 2012)^,^(Sarr, 2012)^,^(Toouli et al., 2002)

## 7. PREVENTION

### 7.1. We recommend perioperative use of diclofenac ad‑ ministered rectally at a dose of 100 mg as a preventive measure in patients at a high risk of PEP. (GRADE 1A) 

ERCP is associated with a risk of PEP in up to 10% of patients, 20% of whom develop moderate or severe forms of PEP.

The high‑risk PEP affects patients with risk factors that can be divided into either patient‑ or procedure‑related.(J‐M et al., 2020)^,^(Mutneja et al., 2021)

The patient‑related PEP risk factors include:

- a history of PEP;
- nondilated common bile duct;
- female sex;
- history of pancreatitis;
- young age;
- obesity;
- congestive renal failure;
- end‑stage renal disease.

The procedure‑related PEP risk factors include:

- difficult cannulation;
- multiple cannulations of the main pancreatic duct;
- administration of a contrast agent into the main pancreatic duct;
- unsuccessful extraction of stones from the bile ducts;
- precut major duodenal papilla;
- pancreatic sphincterotomy.

Of great importance in such situations are preventive measures that include, first and foremost, proper qualification of patients for ERCP. In many cases, alternative imaging studies (EUS, MRCP) can alone contribute to avoiding the risk of PEP in these patients.(J‐M et al., 2020)^,^(Mutneja et al., 2021)

Among the pharmacological prophylaxis methods, a special role is attributed to nonsteroidal anti‑inflammatory drugs (NSAIDs). In a large 2017 meta‑analysis involving a total of 4741 patients, Patai et al(Patai et al., 2017) demonstrated that rectal administration of indomethacin or diclofenac before or after ERCP leads to a significant reduction in the risk of all forms of PEP (relative risk [RR], 0.6; 95% CI, 0.46–0.78, P = 0.0001), including moderate and severe PEP (RR, 0.64; 95% CI, 0.43–0.97; P = 0.0339). Interestingly, administration of these drugs by a route other than rectal was found to have no significant effect on the incidence of complications (P = 0.1507).

On the other hand, Serrano et al(Serrano et al., 2020) performed a meta‑analysis involving a total of 8143 patients subjected to ERCP, with NSAIDs being used be‑ fore or after ERCP in 4020 cases. Finally, a total of 298 cases of PEP were diagnosed in the NSAID group, as compared with 484 in the control group (placebo / no NSAID). The risk of PEP was there‑ fore lower in the NSAID group (risk difference, −0.04; 95% CI, –0.07 to –0.03; number need‑ ed to treat [NNT], 25; P <0.05). In conclusion, NSAIDs were found to effectively prevent mild pancreatitis as compared with placebo (2.5% vs 4.1%; 95% CI, −0.05 to −0.01; NNT, 33; P <0.05); however, the data for moderate and severe PEP were inconclusive. As in the previous study, a favorable prophylactic effect on PEP was observed only following rectal administration (risk difference −0.06; 95% CI, −0.08 to −0.04; NNT, 17; P <0.05) and only for diclofenac or indomethacin being administered at a dose of 100 mg be‑ fore ERCP.

The latest meta‑analysis of the treatment out‑ comes in 7954 patients participating in 26 randomized controlled trials (RCTs), carried out by Kang et al(Kang et al., 2022) in 2023, revealed a superior prophylactic efficacy of diclofenac over indomethacin. The overall incidence of PEP in that study was 7.2% (95% CI, 5.9–8.5), the complication being observed significantly more frequently in the patients receiving indomethacin than in those treated with diclofenac (7.8% vs 3.8%; 95% CI, 6.4–9.3 vs 95% CI, 2.2–5.3; P = 0.009).

Alternative methods of PEP prophylaxis include adequate hydration of the patient during the perioperative period and prophylactic placement of endoprosthesis within the main pancreatic duct, particularly in patients at a high risk of complications. In addition, the use of a guidewire during pancreatic duct catheterization is an independent technical factor that reduces the risk of AP after ERCP. The combination of the above methods has a cumulative effect, significantly reducing the risk of PEP.(J‐M et al., 2020)^,^(Practice Committee et al., 2017)^,^(Yokoe et al., 2015)^,^(Wu et al., 2021)^,^(Zhang et al., 2017)^,^(Zhang et al., 2017)^,^(Phillip et al., 2019)^,^(Radadiya et al., 2022)^,^(Márta et al., 2021)^,^(Akshintala et al., 2021)^,^(Elmunzer et al., 2024)

## 8. CONSERVATIVE TREATMENT: TARGETED THERAPY

### 8.1. We do not recommend the routine use of somatostatin, its analogues, protease inhibitors, antioxidants, glucagon, or insulin in the treatment of AP patients. (GRADE 1A)

Although somatostatin and its analog octreotide inhibit the secretion of pancreatic enzymes, no significant differences in the results of AP treatment as compared with placebo were demonstrated in clinical trials conducted to date. In 2017, Moggia et al(Moggia et al., 2017) confirmed this in an extensive meta‑analysis of as many as 84 RCTs involving a total of 8234 patients. First of all, the early mortality rates were evaluated for AP patients treated with antibiotics, antioxidants, atropine, calcitonin, glucagon, NSAIDs, octreotide, somatostatin, somatostatin with omeprazole, or somatostatin with ulinastatin. The results proved conclusive and showed no effect of these therapies on the mortality rates and other adverse events occurring up to 6 months after the diagnosis of AP. In 2022, Horváth et al(Horváth et al., 2022) performed a me‑ ta‑analysis of studies to compare the effect of somatostatin or ocreotide in combination with ulinastatin. The authors observed a benefit of combination therapy in AP patients as compared with somatostatin / octreotide monotherapy, the benefit consisting in a significant (>70%) re‑ duction in the incidence of acute respiratory dis‑ tress syndrome (ARDS; odds ratio [OR], 0.27; 95% CI, 0.13–0.6) and acute kidney injury (OR, 0.29; 95% CI, 0.09–0.97), and an about 60% reduction in the incidence of multiple organ dysfunction syndrome (MODS; OR, 0.39; 95% CI, 0.2–0.75). A reduction in mortality was also shown in this group of patients, albeit the difference was not significant.

In hypertriglyceridemia‑associated AP, which accounts for about 5% of all cases of the disease, intravenous insulin combined with low‑molecular‑weight heparin (LMWH) can lead to a reduction in TG levels, suggesting that it is theoretically effective in the treatment of this form of AP. Dhindsa et al(Dhindsa et al., 2019) confirmed that combination therapy with insulin and LMWH, as compared with conventional conservative treatment, leads to a reduction in TG levels of 69% vs 63% after 2 days of treatment, and 85% vs 79% after 4 days of treatment, respectively. However, the differences were not statistically significant. For the above reason, this management pathway can be implemented as a safer and, as shown by Piplani et al(Piplani et al., 2024) in their 2024 meta‑analysis, noninferior alternative to plasmapheresis, albeit only in a group of carefully qualified patients.

### 8.2. We propose that AP patients with features of intraabdominal hypertension (IAH) be treated with neostigmin. (GRADE 2B)

One of the common complications of AP is gastrointestinal failure or obstruction leading to increased intra‑abdominal pressure (IAP) and abdominal compartment syndrome (ACS), closely translating to high patient mortality. He et al(He et al., 2023) conducted the first and so far the only meta‑analysis of 6 RCTs to evaluate the efficacy of neostigmin as a prokinetic drug in patients with moderate‑to‑severe AP. The authors showed that the use of neostigmin was associated with significantly shorter times to first bowel movement (mean difference [MD], –1.74; 95% CI, −2.1 to −1.38; P <0.00001) and shorter time to resolution of abdominal symp‑ toms, as compared with the control group (MD,−1.59; 95% CI, −2.07 to −1.11; P <0.00001). At the same time, the researchers observed that the implementation of neostigmin pharmacotherapy resulted in significantly lower IAP values at 24 hours (MD, −8.95%; 95% CI, −13.95 to −3.95; P = 0.0005), which translated to a significantly shorter stay in the intensive care units (ICU; MD: −2.81; 95% CI, −3.75 to −1.87; P <0.00001).(He et al., 2023)

The efficacy of neostigmin in the treatment of AP is still being studied, and a meta‑analysis currently underway by He et al61 aims to assess the di‑ rect effect of this drug on the development of AP within 4 weeks of treatment along with the safe‑ ty of use. This may result in future routine use of neostigmin in the treatment of moderate‑to‑severe forms of AP.

### 8.3. We recommend that proton pump inhibitors (PPIs) be used only as an adjunct to standard AP treatment if significant comorbidities listed as indications for which therapy are present, or if an increased risk of gastro‑ intestinal bleeding or pancreatic pseudocyst (PPC) is recognized. (GRADE 2A)

The available evidence indicates that although treatment with PPIs (to raise the pH of gastric contents) is widely used, it is unlikely to have a beneficial effect on the course of AP itself (including the mortality rate) or on preventing gastrointestinal bleeding in the course of the disease. More‑ over, PPIs can exacerbate pathological processes leading to the development of intra‑abdominal infections. Therefore, routine therapy with drugs that increase gastric pH is not recommended in the cases of pancreatitis.

In patients with severe AP, the released inflammatory mediators (interleukin [IL]‑1β, IL‑6, and tumor necrosis factor α) can cause intestinal dysbacteriosis, which can be further exacerbated by inhibition of gastric acid secretion. Significant increases in duodenal dysbiosis, duodenal bacterial overgrowth, and esophageal thrush were shown when esomeprazole was used in the treatment of AP, as compared with conventional therapy, in a single RCT involving 66 patients. In addition, the released inflammatory mediators may be responsible for excessive permeability of the intestinal mucosa, which, together with bacterial over‑ growth, can lead to bacteremia and local infections and, as a further consequence, to the activation of proinflammatory cytokines and consequently to an even greater intensification of inflammatory processes unfavorable for the prognosis in AP.(Su & Tang, 2021)^,^(Ge et al., 2020)^,^(Watanabe et al., 2017)

A meta‑analysis by Horváth et al,(Horváth et al., 2023) which discussed the potential beneficial effects of PPIs on AP outcomes, was the only publication that revealed a likely positive effect of these drugs on the rate of pseudocysts developing as local AP complications.65 However, this needs to be confirmed in further observations.

## 9. INTENSIVE CARE

### 9.1 We recommend that patients with severe AP and concomitant MODS or at a risk of MODS be treated in the intensive care unit (ICU) setting. The final qualification for ICU treatment shall be based on the current guidelines of the Society of Anesthesiology and Intensive Therapy. (GRADE 1C)

A 2019 multicenter prospective observational study confirmed that any type of organ failure in the course of AP is associated with increased complications and mortality, the most significant determinant of course severity being permanent organ failure.(Sternby et al., 2019) In an observational study, Yu et al(Yu et al., 2023) further demonstrated that the number of organ failures during the course of the disease correlates with mortality. Current guidelines of the Polish Society of Anesthesiology and Intensive Therapy recognize organ failure as a criterion for ICU admission.(Krzych et al., 2022) In contrast, no data are available on the benefits of routine ICU admission in AP patients presenting with no organ failure.

### 9.2. In patients with organ failure, we recommend that basic vital signs be monitored continuously. (GRADE 1C) 

The 5 basic vital signs include temperature, heart rate, blood pressure, respiratory rate, and arterial blood saturation. An observational study from 2022 confirmed the association of deviations in the aforementioned variables with increased mortality rates in patients with life‑threatening conditions.(Candel et al., 2022)

### 9.3. In patients admitted to the ICU for severe AP, we recommend determination of the plasma levels of alanine aminotransferase, aspartate aminotransferase, bilirubin, TG, calcium, phosphorus, creatinine, and urea as well as arterial blood gas levels. (GRADE 1C)

In observational studies published in 2020 and 2023, predictive models for the occurrence of severe AP were established on the basis of the analysis of selected biochemical parameters.(JC et al., 2023)^,^(U et al., 2020) In addition, results of a retrospective study published in 2024 showed a correlation between hypophosphatemia and adverse prognosis and prolonged ICU stays.(Lee et al., 2024)

### 9.4. In order to assess the presence of infected pancreatic necrosis in patients admitted to the ICU for severe AP, we recommend that CRP and procalcitonin levels be determined and an abdominal CT scan be per‑ formed. (GRADE 1B)

According to 2 meta‑analyses published in 2022 and 2024, patients diagnosed with infected pancreatic necrosis in the course of AP presented with increased CRP and procalcitonin levels, as com‑ pared with patients without this complication.(Tarján et al., 2024)^,^(Li et al., 2022) To determine the diagnostic value of these parameters in detecting infected pancreatic necro‑ sis within 72 hours of hospital admission, areas under the curve were calculated for receiver operating characteristic curves, amounting to 0.88 for CRP and 0.86 for procalcitonin. Also, a prospective cohort study published in 2022 confirmed the use‑ fulness of CRP and procalcitonin level determination in the diagnosis of this complication.(Wiese et al., 2022)^,^(Alberti et al., 2022) On the other hand, a prospective RCT (PROCAP) published in 2022 revealed that an antibiotic therapy algorithm based on procalcitonin levels in AP patients facilitates significant reduction in antibiotic use without an increase in the rate of infections during the course of the disease.(Siriwardena et al., 2022)

Infection of pancreatic necrosis can also be diagnosed and monitored by radiological techniques. A cross‑sectional study by Du et al(Du et al., 2022) confirmed high usefulness of repeated multiphase abdominal CT scans in the imaging of infected pancreatic necrosis at different time points in the course of AP.

### 9.5. We recommend IAP monitoring in severe AP patients treated in the ICU setting, particularly in the mechanically ventilated patients. (GRADE 1B)

ACS is a critical complication of severe AP, its risk being particularly high in mechanically ventilated patients. A 2014 systematic review revealed that the incidence rate of this complication in AP patients was as high as 38%. ACS was found to be associated with patient mortality increasing to 49%, as compared with 11% in the non‑ACS group. The syndrome is also associated with a significant increase in the risk of additional organ complications.(, 2014)^,^(A et al., 2011)

### 9.6. We do not recommend intensive fluid therapy due to the increased incidence of respiratory failure and IAH. (GRADE 1B)

### 9.7. We recommend goal‑directed fluid therapy guided by dynamic indices and arterial blood lactate levels. (GRADE 1B)

### 9.8. We suggest using a vasoconstrictor drug (norepinephrine) for arterial hypotension that persists despite adequate fluid therapy. (GRADE 1B)

### 9.9. We recommend fluid therapy based on balanced crystalloid solutions (eg, Ringer lactate). (GRADE 1B)

### 9.10. We do not recommend using 0.9% NaCl solution or synthetic colloids (hydroxyethylated starch, gelatins, dextrans). (GRADE 1B)

Until recently, intensive fluid therapy was part of the canon of therapeutic management of severe AP, justified by the need to ensure adequate visceral perfusion, prevent necrotic lesions, and limit the severity of SIRS. However, recent reports have shown that overly liberal use of fluid therapy not only does not improve patient outcomes but actually increases the risk of complications typical of excessive fluid loading. First and foremost, intensive fluid therapy is associated with an increased risk of IAH, thus contributing to increased disease symptoms and prolonged hospitalization. In view of the above, a regimen of limited fluid supply, that is, goal‑directed fluid therapy, should be pursued, with monitoring of the patient’s volemia in real time using available clinical parameters or hemodynamic methods. One of the recommended ways to prevent fluid overload, especially in the resuscitation phase, is early implementation of vasoconstrictors (norepinephrine). Recently published meta‑analyses comparing the outcomes of AP treatment depending on the type of crystalloid solution and fluid therapy protocol revealed that the use of balanced fluids is associated with reduced severity of SIRS, reduced duration of hospitalization, lower frequency of ICU admissions, and a reduction in the overall number of complications.(E et al., 2022)^,^(Martino M et al., 2021)^,^(Wang et al., 2024)^,^(Aziz et al., 2021)

### 9.11. In patients diagnosed with AP, we recommend multimodal pain therapy based on nonopioid and opioid analgesics. We do not recommend intravenous infusions of local anesthetic agents as a means of pain management. (GRADE 1B)

Pain management is the highest priority in AP. Pain is present in nearly all patients with AP, and is the main reason for presentation at the physician’s office.(Petrov & Yadav, 2019) Despite relative contraindications to the use of opioids (morphine) still in place in clinical practice, they continue to be considered the first line of analgesia in AP patients due to their sphincter of Oddi spasm–producing and immunosuppressive effects, which are extremely important in the initial phases of the disease, as well as to their permeability through the intestinal barrier. The results of the most recent 2023 meta‑analysis of 8 RCTs are unequivocal and point to the equal analgesic potential of opioid and nonopioid drugs. No statistical differences were observed in terms of adverse effects, pain severity, requirement for coanalgesia, length of hospital stay, and mortality in both the adult and pediatric populations, with the main opioid‑related adverse effects being nausea, vomiting, and brief episodes of hypotension.(Almulhim et al., 2023)^,^(Barlass et al., 2018)

In a 2022 meta‑analysis on pain management in the first phase of AP, Thavanesan et al(Thavanesan et al., 2022) identified 12 RCTs comparing 7 analgesic drugs and techniques, including opioids, NSAIDs, metamizole, local anesthesia, epidural anesthesia, and paracetamol vs placebo. In all the examined modalities, therapeutic outcomes, as measured by the visual analogue scale (VAS), showed an overall improvement from baseline by day 2. Epidural anesthesia appeared to provide the greatest efficacy in the first 24 hours, while shown to be equivalent to opioids after 48 hours. During the first 24 hours, NSAIDs provided pain relief similar to that obtained using opioids (as measured by VAS). Interestingly, placebo was also shown to be equivalent to other modalities in the early phase, while quick‑ ly reaching plateau. Eventually, local anesthetics had the lowest overall therapeutic efficacy. However, the observations were fraught with an error due to the significant statistical and methodological heterogeneity of the analyzed RCTs.

### 9.12. In critically ill patients, we recommend that thoracic epidural anesthesia be used as an alternative pain treatment. (GRADE 1B)

The data reported in the literature published in recent years have been inconsistent. Some retrospective studies and prospective RCTs suggested improved prognosis for AP patients in critical condition receiving continuous epidural analgesia in the thoracic spine.(Jabaudon et al., 2018)^,^(Sadowski et al., 2015) Likewise, in a meta‑analysis of 9 studies involving a total of 2006 patients, Jabaudon et al(Jabaudon et al., 2023) showed that this modality was both effective and safe in relieving pain while additionally reducing ultimate mortality rates by improving the blood supply to the pancreas. Unfortunately, the prospective 2023 EPIPAN multicenter study in AP patients treated in the ICU setting found no differences between the study group (which received epidural analgesia) and the control group, either in terms of the number of complications or even in terms of the effect on pain severity.(Jabaudon et al., 2023)

## 10. INTRA‑ABDOMINAL HYPERTENSION

It is estimated that a vast majority (up to 60%) of patients with AP develop IAH at some point of the disease course, while >30% of patients develop ACS.(Jena et al., 2023) Despite that, the phenomenon remains significantly underestimated to this day. Pancreatitis alone—through accompanying visceral edema, bowel obstruction, peripancreatic fluid accumulation, ascites, or retroperitoneal edema—can be a risk factor for IAH. In addition, the routine administration of very large amounts of crystalloids and colloids in the initial phase of AP treatment significantly affects the risk of IAH, which may need to be managed on an ad hoc basis, especially if it exceeds 25 mm Hg.

The pathological IAP elevation results in a change in the abdominal perfusion pressure (the difference between mean arterial pressure and intra‑abdominal pressure). Lowered abdominal perfusion pressure of <50 mm Hg is associated with a high mortality rate and requires emergency interventions.

The following symptoms may be indicative of IAH:

- abdominal bloating not yielding to hand pressure, possibly accompanied by tenderness;
- worsening oliguria;
- increasing respiratory failure;
- hypotension;
- tachycardia;
- jugular vein distention;
- peripheral edema;
- acidosis.

### 10.1. We recommend that IAP be measured and IAH treatment be implemented whenever it is suspected. (GRADE 1C)

Aside from its prophylactic significance, the measurement of IAP in the course of severe AP is important for therapeutic decisions. In an RCT by Xie et al,(Xie et al., 2021) patients with IAP ≥20 mm Hg could be expected to derive therapeutic benefit from the use of continuous veno‑venous hemofiltration. On the other hand, the effectiveness of such treatment was not been confirmed when the IAP values did not exceed 20 mm Hg. Therefore, IAP monitoring may be a key element in deciding whether and when to initiate continuous veno‑venous he‑ mofiltration, which might contribute to additional therapeutic benefits in a group of AP patients.(Xie et al., 2021)

### 10.2. Once IAH is diagnosed, we first recommend placing the patient in a supine position, their legs slightly bent, followed by the delivery of appropriate analgesia and sedation, and decompression of the stomach (using a nasogastric tube), bladder (using a catheter), and colon (using a drain inserted into the rectum). (GRADE 1C)

### 10.3. We recommend paracentesis / transcutaneous drainage in the cases of IAH‑complicated AP accompanied by ascites. (GRADE 2C)

IAH induced by acute‑phase fluid reservoirs may entail negative consequences in AP patients. Percutaneous drainage of such spaces can lower this pressure by up to 40%, leading to improved prognosis, as documented in an observational study by Singh et al.(Singh et al., 2020) Similar conclusions were drawn by Wen et al(Wen et al., 2023) on the basis of a retrospective study: the researchers observed a significant positive effect of paracentesis on the mortality rate and the extent of organ failure in the course of AP. In contrast, emphasizing the importance of surgical decompression on the basis of their comparative study, Cheatham and Safcsak(Cheatham & Safcsak, 2011) concluded that if ≥1000 ml of fluid cannot be drained from the peritoneal cavity to lower the IAP value by ≥9 mm Hg within the first 4 hours, decompressive laparotomy should be performed.

### 10.4. In the absence of the therapeutic effect of non‑ surgical procedures used in the treatment of IAH/ACS, we recommend that minimally invasive techniques and procedures be used; open abdominal treatment should follow if the former are ineffective. (GRADE 2C)

Surgical decompression is an effective intervention capable of promptly reducing the IAP. It is recommended in patients with IAH >25 mm Hg, but may also be considered in patients with IAH >15 mm Hg, provided they develop progressive cardiorespiratory failure or have failed previous nonsurgical treatment.(Burch et al., 1996)

However, high‑quality scientific data are lacking to establish the optimum timing and method of surgical decompression. In a review arti‑ cle based on a simple analysis of the literature, Mancilla Asencio and Berger Fleiszig(C & Z, 2022) propose that minimally invasive techniques be used as the first choice.(C & Z, 2022) Further controlled prospective studies are undoubtedly needed in this area.(Nasa et al., 2023)

## 11. ANTIBIOTIC PREVENTION AND TREATMENT

### 11.1. We do not recommend prophylaxis with anti‑ microbial drugs, regardless of the severity of AP. (GRADE 1A)

To date, scientific data on the efficacy of antibiotic prophylaxis for AP have been quite clear. Although a meta‑analysis of RCTs, published in 2022, showed a reduction in the overall risk of infection in AP patients receiving such prophylaxis, this effect was only due to a reduction in the risk of extrapancreatic infection, the most significant risks related to the infection of acute phase reservoirs or pancreatic necrosis remaining unaffected. In addition, due to the risk of multidrug‑resistant strain selection and invasive candidiasis, routine use of antibiotic prophylaxis cannot be recommended.(Poropat et al., 2022)^,^(Timmerhuis et al., 2023)^,^(Horibe et al., 2019)

However, daily clinical practice has deviated from the long‑standing recommendation not to use prophylactic antibiotics in AP patients; as shown by the studies, this is a worldwide problem. This may be due to some meta‑analyses published after the year 2000 suggesting a beneficial effect of broad‑spectrum antibiotic prophylaxis in patients with severe necrotizing AP. However, the above observations were made on very small groups, and subsequent publications did not confirm the efficacy of such treatment.(Sharma & Howden, 2001)^,^(Poropat et al., 2019)

In recent years, studies focusing on the use of antibiotic prophylaxis using drugs that are not absorbed from the gastrointestinal lumen (rifaximin) have also been conducted, and showed a reduction in the duration of hospitalization following the administration of such agents.105 However, RCTs on this group of medications are required before recommending their routine use in AP patients.

In conclusion, prophylactic administration of antibiotics is not recommended; this applies to cases of both mild and moderate or severe AP. The absence of recommendation for antibiotic prophylaxis also applies to special clinical populations (children, pregnant women, immunosuppressed patients).(Venkatesan, 2008)^,^(Moggia et al., 2017)^,^(Wilkins & Sequoia, 2017)^,^(Berg FF & MA, 2023)^,^(Takada et al., 2022)^,^(Dellinger et al., 2007)^,^(García‐Barrasa et al., 2009)^,^(Benson et al., 2023)^,^(Abu‐El‐Haija et al., 2018)

### 11.2. We do not recommend the use of pro‑ or sym‑ biotics, regardless of the severity of AP. (GRADE 1B)

The routine use of pre‑, pro‑, or symbiotics cannot be recommended at this time due to the lack of unequivocal confirmation of their beneficial effect on the course of AP. Admittedly, in a single meta‑analysis of 6 RCTs, the length of hospital stay of patients in the study group who received the aforementioned preparations was found to be shorter (weighted MD, −5.49 days; 95% CI, −10.4 to −0.58; P = 0.01; the control group received placebo or standard treatment without probiotics); however, no significant effect was observed for the intake of these agents on the reduction in mortality rate or the risk of organ failure.(Malik et al., 2023)^,^(Gao et al., 2023)^,^(Yu et al., 2021)

Moreover, it should be kept in mind that as early as 2008, the results of an RCT by Besselink et al(Besselink et al., 2008) unequivocally showed that the use of probiotics in severe AP not only does not reduce the risk of infectious complications but actually increases the mortality rate in this group of patients.(Besselink et al., 2008)

### 11.3. We do not recommend the use of antimicrobial therapy to treat infected pancreatic necrosis on the basis of clinical symptoms and CRP measurements alone. Diagnosis of the infection of pancreatic necrosis should be based on imaging studies (eg, contrast‑enhanced abdominal CT scans). However, we recommend that procalcitonin levels be monitored to optimize the use of antimicrobial drugs. (GRADE 1A)

The decision to initiate pharmacological treatment of septic complications of AP should be based on certain clinical data, that is, the results of imaging studies (indirect features of infected necrosis—gas bubbles, features of abscess— on CT scans), microbial blood cultures, examination of the purulent contents or necrotic biopsy material, and inflammatory marker (procalcitonin) levels. Pyrexia alone is not a sufficient indication for the implementation of antibiotic therapy. Due to the fact that elevated CRP levels are observed in the course of SIRS regardless of the status of infectious complications, CRP is not a good marker of infection. In contrast, high levels of both inflammatory markers sustained over a period of >72 hours are a reliable predictor of infection, as documented in a 2024 meta‑analysis by Tarjan et al.(Tarján et al., 2024) At the same time, no data on the utility of IL‑6 in the diagnosis of infectious complications of AP are available.(Alberti et al., 2022)^,^(Siriwardena et al., 2022)^,^(Saeed et al., 2019)

### 11.4. We recommend that the treatment of infected necrotic foci be started with antimicrobial drugs rather than a surgical intervention (step 0). (GRADE 1C)

Antibiotic therapy for infectious complications of AP—both extrapancreatic infections and lo‑ cal septic complications in the form of infected fluid reservoirs or infected necrosis—remains the treatment of choice and usually becomes part of a broader treatment strategy combined with drainage procedures. In at least some cases, antibiotic therapy alone may prove to be effective in the treatment of infected pancreatic necrosis, constituting step 0 of the step‑up approach (see below).(Boxhoorn et al., 2021)^,^(Obeid et al., 2023)^,^(Al‐Sarireh et al., 2018)

### 11.5. If the infection of pancreatic necrosis is confirmed, we recommend that empirical antimicrobial therapy targeting the Enterobacteriaceae, Enterococcus faecium, and Pseudomonas aeruginosa strains be implemented. While empirical treatment should be modified according to the results of microbial cultures, obtaining the material for microbiological examinations is not necessary for the initiation of treatment. (GRADE 1C)

If infectious complications are strongly suspected, a reliable microbiological culture of infected tissue or blood should be obtained. This is related to the observation that up to 50% of cultures obtained from patients with septic complications of AP present with no sensitivity to the previously used empirical antibiotic therapy. 

Due to the usually severe condition of the patient at the time of the diagnosis of septic complications of AP, empirical intravenous antibiotic therapy should be implemented without un‑ due delay using broad‑spectrum agents capable of penetrating into the necrotic environment. It is important to keep in mind that in most cases (60%–87%), the necrotic tissue or acute phase reservoirs are infected with a single strain, pre‑dominantly a gram‑negative strain.(Lu et al., 2019)

According to the statistics, about 30% of AP patients develop infectious complications of extrapancreatic location. The most common of these is pneumonia.(Brown et al., 2014)

Most of the suggestions for empirical anti‑ biotic selection formulated to date were based on the results of pharmacodynamic analyses rather than on comparative clinical trials that could result in the recommendation of a specific active substance. As a result, carbapenems or fluoroquinolones and metronidazole(Hackert & Büchler, 2016)^,^(Baron et al., 2020) were the most common choice to date. However, in light of recent reports describing an increased incidence of chronic neuropathic pain following the use of fluoroquinolones, carbapenems (meropenem, ertapenem) remain the drugs of choice. As for this group of antibiotics, imipenem with cilastatin is unlikely to be recommended due to potential problems in achieving adequate antibacterial concentrations in the infected necrotic foci and acute phase reservoirs.(He et al., 2016) For the same reason, despite identification of vancomycin‑susceptible gram‑positive bacterial cultures, vancomycin should not be included in therapy due to its very poor penetration into the infected necrotic foci and acute phase reservoirs.(He et al., 2016) An antibiotic from the oxazolidinone group (such as linezolid) seems to be a better choice. Notably, clindamycin and piperacillin with tazobactam also show good penetrating properties in such cases.(Mowbray et al., 2018)^,^(Mourad et al., 2017)

### 11.6. It is not possible to recommend specific anti‑ microbial drugs to be used in empirical treatment of infected pancreatic necrosis. The choice should be based on local infection maps, drug resistance prevalence, and the pharmacokinetic / pharmacodynamic profile (PK/PD) of the antimicrobial drug. (GRADE 1C)

Despite the results of microbiological examination indicating the sensitivity of microorganisms to the chosen antibiotic, it should be borne in mind that due to the nature of the disease, specific antibiotics may show inadequate penetration into infected necrotic foci and peri‑ pancreatic reservoirs. Dose selection should not be guided solely by data from the summary of product characteristics.

### 11.7. We recommend that the antimicrobial drugs be dosed according to their PK/PD profiles. (GRADE 1C)

Significant changes in the pharmacokinetics and pharmacodynamics of drugs are observed in critically ill patients. This is due to a number of hemodynamic and metabolic disorders. These include an increase in extravascular water volume, hypoalbuminemia, or increased endothelial permeability of blood vessels. Failure to take into account the pathophysiological changes occurring in this group of patients may result in inappropriate antibiotic dosage and, consequently, in pharmacotherapy failure, delayed clinical response to pharmacotherapy, or development of resistance to the antimicrobial agent used.(Timsit et al., 2019)

### 11.8. We recommend that empirical antifungal therapy be followed only in patients at a high risk of invasive fungal infection or those with confirmed fungal infection. (GRADE 1B)

As of present, no data are available on the appropriateness of empirical antifungal therapy being implemented in all AP patients, regardless of the severity of disease symptoms. It seems that only patients at a high risk of invasive fungal infection (most commonly of Candida spp. etiology) may benefit from the implementation of empirical therapy. The risk factors for Candida spp. infection include multiple yeast colonization sites, immunosuppression, high APACHE score, prolonged ICU stay, central vascular accesses, parenteral nutrition, intestinal anastomotic leaks, abdominal or biliary surgical interventions, and acute renal failure and dialysis.(Timsit et al., 2019)^,^(Timsit et al., 2016)^,^(Jaber et al., 2022)

## 12. THROMBOPROPHYLAXIS / ANTICOAGULATION

### 12.1. In all patients hospitalized for AP, we recommend that the risk of venous thromboembolism (VTE) be assessed using dedicated scales (eg, the Padua scale for conservatively treated patients, the Caprini scale for surgically treated patients). In the cases of a moderate‑to‑high risk of VTE, we recommend that thromboprophylaxis be implemented according to current guidelines. (GRADE 1B)

As with all motion‑limiting diseases, patients with AP, especially those with a severe course of the disease, are at a significant risk of VTE. AP can also be complicated by visceral vein thrombosis, which can adversely affect the final prognosis. However, the available results of RCTs evaluating the impact of LMWH treatment in AP patients are inconsistent. The reports published to date are insufficient to assess the benefit / risk ratio of high‑dose anticoagulant prophylaxis being started in AP patients at a high risk of visceral vein thrombosis or of the implementation of treatment in patients with confirmed visceral vein thrombosis.(Jaber et al., 2022)^,^(He et al., 2023)^,^(Patil et al., 2022)^,^(Gandikota et al., 2021)^,^(Slawinski & O’Reilly, 2017)

### 12.2. We propose that prophylactic LMWH doses be used in all cases of moderate‑to‑severe forms of AP, provided the absence of specific contraindications or increased risks associated with such treatment. (GRADE 1B)

In 2024, a systematic review with meta‑analysis of 8 RCTs and 5 nonrandomized trials (in a total of 13 709 patients) confirmed that LMWH treatment can favorably affect the prognosis for patients with moderately severe or severe AP. Moderate‑ to high‑strength evidence suggests that heparins used early in the course of the disease may improve the prognosis in the form of a reduced risk of death (RR, 0.44; 95% CI, 0.31–0.64; P <0.0001, I2 = 51%), organ failure (RR, 0.67;95% CI, 0.48–0.93; P = 0.02; I2 = 78%), and vascular thrombotic complications (RR, 0.43; 95% CI, 0.31–0.61; P <0.00001; I2 = 0%). In addition, scientific data of low‑to‑moderate quality support the conclusion that LMWH combined with conventional therapies can reduce the incidence of hemorrhagic complications and shorten the duration of hospital stays. In light of these findings, heparin inclusion can be recommended in the management regimen for moderate‑to‑severe AP, keeping in mind the balance between efficacy and therapeutic safety, and assuming that prophylactic doses ap‑ pear more effective than therapeutic ones.(Podda et al., 2024) Similar conclusions were arrived at by Patil et al(Patil et al., 2022) and Gandikota et al,(Gandikota et al., 2021) authors of a published RCT and a prospective study conducted at a referral center. The researchers found that the administration of prophylactic‑dose LMWH shortens the length of hospital stay and reduces the severity of the dis‑ ease, the risk of pancreatic parenchymal necrosis, and mortality.

## 13. NUTRITION / NUTRITIONAL TREATMENT

Due to the increased catabolism and reduced gastrointestinal capacity, patients with severe AP

require early inclusion of artificial enteral or intravenous nutrition. Enteral nutrition refers to both intestinal and gastric feeding.

### 13.1. We recommend that nutrition in an amount tailored to the metabolic capabilities of the AP patient be implemented as soon as the prognostic factors of a severe course are established, cell perfusion is ensured, and severe water‑electrolyte and acid‑base disorders are controlled. (GRADE 1A)

Through the increased catabolism, severe AP quickly leads to malnutrition and translates into a high risk of associated complications. The above posi‑ tion has been presented by the European Society for Clinical Nutrition and Metabolism (ESPEN) and the World Society of Emergency Surgery (WSES).(Arvanitakis et al., 2020)^,^(Leppäniemi et al., 2019) In‑hospital mortality, sepsis, septic shock, and respiratory failure rates are higher in malnourished patients.(Le et al., 2023) Reliable scientific data indicate that early initiation of artificial nutrition (within 48 hours of hospital admission) reduces the risk of developing MODS.(Feng et al., 2017)

The essence of nutrition consists in the delivery of macronutrients to the cell. Disruption of cell per‑ fusion (shock) is therefore a natural contraindication to nutrition in any form. Similarly, severe water‑electrolyte and acid‑base imbalances are associated with abnormalities in intracellular metabolism.(Kott et al., 2019)^,^(Al‐Jaghbeer & Kellum, 2015) Thus, the provision of macronutrients needs to be started only after these disorders are completely or partially (if complete equilibration is not achievable at a certain stage of the disease) controlled. Feeding should be started gradually. Enteral nutrition is initiated at a rate of 20 ml/h, with subsequent increase in the flow rate by 20 ml/h every 12 hours and constant monitoring of tolerance. Similarly, parenteral nutrition should be started at a volume of calculated partial requirement (eg, 50%) and increased on subsequent days, as guided by relevant biochemical parameters.

### 13.2. We recommend gastrointestinal feeding as the preferred method for the delivery of nutrients. (GRADE 1A)

Enteral nutrition is a very important factor in stabilizing the intestinal barrier, thus reducing the risk of bacterial translocation and, consequently, infection of pancreatic necrosis. It is also associated with a lower risk of other infectious complications and a significantly lower risk of exceeding metabolic limits, or overfeeding. Properly administered enteral nutrition (properly selected quantities and type of medical diet, infusion method, and access care) is safe and effective for patients with severe AP.(Elke et al., 2016)^,^(Yi et al., 2012)^,^(Bakker et al., 2014)^,^(Preiser et al., 2021)^,^(Lee et al., 2022)^,^(Song et al., 2018)

### 13.3. When gastric motor function is preserved, we propose that intragastric nutrition be used, the enteral nutrition—administered via the ligament of Treitz—being recommended for patients with gastric stasis. (GRADE 1B)

Insertion of a gastric feeding tube is easier and more stable than the insertion of an intestinal feeding tube. In their meta‑analysis, Dutta et al(Dutta et al., 2020) failed to demonstrate any superiority of intragastric feeding over enteral feeding in a group of AP patients. On the other hand, impaired gastric emptying is associated with gastric stasis and, consequently, with a reduction in the potential for supply and assimilation of nutrients administered by this route. Several observational studies and RCTs have documented that nutrition administered from a tube placed out‑ side the ligament of Treitz (naso‑jejunal tube or nutritional microjejunostomy) in patients with preserved bowel function allows for continued successful feeding.(Dutta et al., 2020)^,^(Li et al., 2019)^,^(Gungabissoon et al., 2015)^,^(Qiu et al., 2017) Wang et al(Wang et al., 2024) performed a meta‑analysis comparing the effects of both feeding routes for nutrition initiated in the first 48 hours of the disease to find no significant differences in terms of the need for surgical intervention, parenteral nutrition, or success rates of feeding procedures. Instead, a significantly higher incidence of diarrhea (RR, 2.75; 95% CI, 1.21–6.25; P = 0.02) and pain (RR, 2.91; 95% CI, 1.50–5.64; P = 0.002), as well as a higher risk of infection (6.67% vs 3.33%; P = 0.027) and incidence of MODS were observed in the enteral feeding group. Eventually, the aforementioned analysis showed that both enteral and gastric nutrition are valid therapeutic options for the early treatment of AP and present with no significant differences in terms of overall mortality.

### 13.4. We recommend that supplemental or total parenteral nutrition be used in patients with limited or total gastrointestinal failure or IAH preventing the delivery of full / minimal enteral nutrition. (GRADE 1B)

Gastrointestinal failure, as a component of MODS resulting from the multidirectional effects of vasoactive drugs, IAH, or peritonitis, can partially or completely limit the supply of nutrients via the gastrointestinal route. Therefore, parenteral nutrition can become an effective option for supplementing the patient’s nutritional needs in the course of the disease. Based on a meta‑analysis of 5 RCTs, Alsharif et al(Alsharif et al., 2020) confirmed that supplemental intravenous feeding reduces the risk of septic complications and mortality as compared with ineffective feeding by the enteral route alone. On the other hand, to be maximally effective, intravenous feeding should be delayed until at least the fourth day after the start of enteral feeding to ensure sufficient metabolic effect and reduce the need for nutrients delivered by the intravenous route.(Alsharif et al., 2020)^,^(Ridley et al., 2018)^,^(Luo & Qian, 2020)

### 13.5. We recommend that the individual components of the feeding mixture be selected according to metabolic limits. (GRADE 1C)

The failure to balance the daily nutrient intake and unreflective ordering of large doses of parenteral nutrition, particularly in critically ill patients, is a significant clinical problem in modern intensive care. Liver damage may quickly occur in such cases, along with the increase in the risk of infectious complications, the duration of ventilation replacement, renal replacement therapy, and the total length of ICU stay. This was best con‑ firmed in a study by Casaer et al,(Casaer et al., 2011) where the patients received intravenous nutrition at levels as high as 30 kcal/kg of body weight; despite the authors’ incorrect interpretation of their own study results, the report remains available in the literature as a warning to those attempting to follow such regimens. On the one hand, it should always be borne in mind that the sup‑ ply of water, electrolytes, and macronutrients should be adjusted on a daily basis according to the patient’s clinical condition, fluid balance, and blood biochemistry results. On the other hand, due to the lack of good indicators, the administration of vitamins and trace elements should be based on fixed daily doses.(Casaer et al., 2011)^,^(Tanaka et al., 2020)

## 14. DIAGNOSIS OF INFECTED PANCREATIC NECROSIS

### 14.1. We do not recommend routinely performing fine‑needle aspiration biopsy to confirm infection, as it frequently leads to secondary infection of the necrotic tissue. (GRADE 1C)

### 14.2. For the diagnosis of infected pancreatic necrosis, we recommend reliance on indirect evidence, that is, the presence of gas bubbles outside the gastrointestinal lumen on multiphase abdominal and pelvic CT scans, after exclusion of gastrointestinal perforation. (GRADE 1C)

Infection of pancreatic necrosis in the course of AP usually occurs through translocation of intestinal bacterial flora from the gastrointes‑ tinal tract. For this reason, the most common pathogens in these cases include Escherichia coli, Klebsiella pneumoniae, Enterococcus faecalis, Staphylococcus aureus, Pseudomonas aeruginosa, Proteus mirabilis, and Streptococcus spp.(Stamatakos et al., 2010)^,^(Olson & abscess, 1989)^,^(Jagielski et al., 2019)^,^(Wroński et al., 2015)

When infection of pancreatic necrosis or peri‑ pancreatic tissues is suspected, diagnostic and therapeutic management can be based on indi‑ rect or direct evidence of infection as a rationale for the implementation of intravenous antibiotic therapy. Direct confirmation of peripancreatic necrotic tissue infection can only be provided by a positive culture of the contents collected from the reservoir lumen in a fine‑needle aspiration biopsy. However, this method is no longer recommended in routine management, as it has often led to secondary infections of such areas. For this reason, therapeutic decisions should be based on indirect symptoms whenever possible, until a reliable culture result is obtained for biological material collected by other routes (eg, endoscopic intervention, percutaneous drainage, or surgery). Indirect evidence of the infection of pancreatic / peripancreatic necrosis is most frequently provided by the presence of gas bubbles outside the gastrointestinal lumen on multiphase abdominal and pelvic CT scans, after gastrointestinal perforation has been excluded. Other evidence may be provided by prolonged or new symptoms of SIRS observed 7 days after the onset of AP.(Crockett et al., 2018)

## 15. DIAGNOSIS AND CLASSIFICATION OF AP SEQUELAE

### 15.1. We propose that the 2012 revision of the Atlanta Classification be used to describe the complications and sequelae of AP. (GRADE 1C) 

Moderate‑to‑severe clinical course of AP is associated with a high risk of local complications and organ failure that significantly increase patient mortality.

The most common local complications of AP include post inflammatory pancreatic and peripancreatic fluid reservoirs. Acute peripancreatic fluid reservoirs can form already at the early stag‑ es of the interstitial edematous form of AP, naturally transforming into PPCs after about 4 weeks of illness; most of these early‑phase reservoirs resolve spontaneously without the need for interventional treatment. In contrast, PPCs, which are late‑stage reservoirs, are characterized by an already well‑developed wall, the thicker and the better structured, the longer the reservoir persists.

In the course of necrotic AP, necrosis develops within the organ itself and / or within peripancreatic tissues. During the first 4 weeks of the disease, pancreatic fluid reservoirs are referred to as acute necrotic reservoirs. They generally take the form of poorly confined fluid spaces containing a large amount of dead tissue within their lumen. Nearly half of these regress spontaneously, while the remainder evolve into walled‑off pancreatic necrosis after ≥4 weeks. According to the definition, walled‑off pancreatic necrosis is a fluid reservoir containing solid and liquefied dead tissue, the degree of liquefaction depending on the time that has passed since the onset of the disease.

The second most common group of local complications of AP are vascular complications. First among these are splenic vein thrombosis and portal vein thrombosis, which can lead to the development of portal hypertension, resulting in esophageal varices and gastric fundic varices.(Banks et al., 2013)^,^(Boxhoorn et al., 2020)^,^(Sarr, 2012)^,^(Jagielski et al., 2019)^,^(Wroński et al., 2015)^,^(Thoeni, 2012)

## 16. DIFFERENCES IN THE MANAGEMENT STRATEGY AND PRACTICE DEPENDING ON THE ETIOLOGY OF AP

In any case of AP, the cause of the disease should be clarified as soon as possible, as it determines the subsequent management, that is, the treatment and the pattern of follow‑up examinations. 

When determining a management strategy, we also recommend referring to the Guidelines for the Use of Laparoscopy in Acute Abdominal Diseases – the Position Statement of the Expert Group of the Association of Polish Surgeons.(Sobocki et al., 2023)

### 16.1. In the case of biliary AP with cholangitis, we recommend that ERCP with endoscopic sphincterotomy and evacuation of deposits be performed within 24 hours. (GRADE 1A)

### 16.2. In the case of biliary AP with persistent biliary obstruction and no cholangitis, we recommend that ERCP with endoscopic sphincterotomy and evacuation of deposits be performed within 72 hours. (GRADE 1A)

Determining the optimum timing for ERCP in the cases of acute cholangitis in the context of potential clinical benefits was the objective of a cohort study conducted by Seo et al. The analysis of a database containing the results of >91 000 patients treated for acute cholangitis clearly showed that ERCP performed during the first 72 hours was associated with a relatively low mortality rate which increased significantly when the procedure was performed on subsequent days (ie, days 4–7). Also, a retrospective study involving >4500 AP patients with concomitant cholangitis symptoms revealed that early (<48 h) ERCP was associated with reduced early mortality rates (up to 30 days). Finally, a meta‑analysis encompassing the results of treatment of 1639 patients with biliary AP, carried out by Tang et al., showed significantly lower complication rates, better pain control, and shorter times to resumed peristalsis, normalization of amylase activity, and ultimately a shorter duration of hospital stays in the group of patients undergoing endoscopic treatment.(Mulki et al., 2019)^,^(Mukai et al., 2023)^,^(Seo et al., 2020)^,^(Tang et al., 2022)^,^(Tenner et al., 2024)^,^(Burstow et al., 2015)^,^(Coutinho et al., 2018)

### 16.3. We do not recommend performing ERCP to treat biliary AP in patients with cholecystolithiasis presenting with no features of persistent biliary obstruction or cholangitis. (GRADE 1B)

As shown in a multicenter RCT conducted by the Dutch Pancreatitis Study Group,(Schepers et al., 2020) urgent ERCP in patients with suspected severe pancreatitis with cholelithiasis but no signs of cholangitis did not reduce the rates of major complications or mortality, as compared with the conservatively treated group. These results confirm that such treatments only benefit patients with symptoms of cholestasis and / or cholangitis. These above observations were also confirmed by Shrestha et al,(Shrestha et al., 2022) who carried out a meta‑analysis of studies on the effectiveness of ERCP in AP patients. In the case of biliary AP without concomitant cholangitis, emergency endoscopic surgery did not reduce mortality or the risk of complications, as compared with conservative management.

### 16.4. Cholecystectomy is indicated in the treatment of AP in patients with confirmed cholecystolithiasis. Perioperative risks in AP as well as risks of complications of cholelithiasis should be taken into account when deciding on the timing of the procedure. We propose that cholecystectomy be performed in patients with mild AP against the background of cholecystolithiasis during the same hospital stay. (GRADE 1A)

As highlighted by Blundell et al,(Blundell et al., 2023) deferral of cholecystectomy in the cases of benign biliary AP results in increased rates of readmissions, indications for ERCP, and conversion of laparoscopic to open surgeries. The authors argue that cholecystectomy should be performed during the first hospitalization and, if that is not possible, within the next 30 days. An important limitation in terms of the need to perform ERCP in patients with benign biliary AP undergoing early cholecystectomy, that is, the need for the procedure be performed within 24 hours of hospital admission, has also been indicated in a study by Mueck et al.(Mueck et al., 2019) Early cholecystectomy also shortens the duration of hospitalization and reduces the risk of some complications. Yuan et al(Yuan et al., 2021) and Dai et al(Dai et al., 2021) carried out independent meta‑analyses to infer that postponing ECRP may be associated with a significant increase in the risk of recurrent AP, biliary incidents, with risks comparable to those associated with surgical gallbladder removal alone (see eg, the PONCHO RCT).(Yuan et al., 2021)^,^(Dai et al., 2021)^,^(Costa DW et al., 2015)^,^(Takada et al., 2013)

### 16.5. In the case of gallstone‑related AP involving the development of peripancreatic fluid reservoirs, we recommend that cholecystectomy be postponed until resorption/stabilization of the reservoir and resolution of inflammation. (GRADE 1B)

Determining the optimum timing for cholecystectomy in moderate‑to‑severe biliary AP is still a matter of clinical debate. Some weak evidence indicates that deferring the surgery for ≥6 weeks may be associated with reduced mortality and fewer complications. The same delay in the procedure also appears to be important in patients with AP complicated by the presence of a peripancreatic reservoir.(Hughes & Morris‐Stiff, 2020)^,^(Nealon et al., 2004) Schepers et al(Schepers et al., 2020) carried out a multicenter RCT to demonstrate the appropriateness of deferring cholecystectomy in these cases until the acute inflammation has resolved and the fluid has resorbed or the size of the reservoir has completely stabilized.

### 16.6. For significant hypertriglyceridemia, we suggest insulin infusion, especially in diabetic patients, and for persistently high TG values in patients with severe (>11.3 mmol/l) or very severe (>22.4 mmol/l) hypertriglyceridemia, we recommend considering therapeutic plasma exchange, especially if there is no response to conservative treatment (fibrates, heparin, insulin). (GRADE 2C)

The etiology of AP associated with hypertriglyceridemia justifies the implementation of causal treatment in addition to classic symptomatic management. Fibrates are among the treatments of choice in the aforementioned cases. In addition, insulin can reduce serum TG levels by as much as 50%–75% within 3 days of use, with LMWH also recommended in such situations. However, at present, the literature, does not provide strong evidence to justify the implementation of specific AP therapies. Only on the basis of observational studies can it be concluded that in the case of serum TG levels being persistently high (>1000 mg/dl) despite the initiated treatment, hemoperfusion or plasmapheresis with double filtration can provide additional positive therapeutic effects. For this reason, individualized management is recommended in any such case.(Afari et al., 2013)^,^(Coskun et al., 2015)^,^(Wang et al., 2023)

## 17. QUALIFICATION FOR SURGICAL TREATMENT: INTERVENTION TIMING AND PURPOSE

When qualifying patients for surgical treatment, the Guidelines for the Use of Laparoscopy in Acute Abdominal Diseases – the Position

Statement of the Expert Group of the Association of Polish Surgeons should also be followed.(Sobocki et al., 2023)

### 17.1. On the basis of the available data, no recommendations can be made regarding the timing of surgical intervention in severe forms of AP, regardless of the type of such intervention (open, minimally invasive, or endoscopic). (GRADE 2B)

When making strategic therapeutic decisions, one should be guided by the principle that the current dynamic clinical condition of the patient should always be the first determinant of management strategy.

The choice of the optimum timing of intervention depends on the stage of severe AP:

1. early acute phase (≤4 weeks since the on‑ set of disease symptoms);
2. late phase (>4 weeks since the onset of disease symptoms).

SIRS‑associated MODS frequently develops during the first / second week of the disease. At this time, conservative treatment and monitoring of the patient’s condition are mainly recommended, preferably in the ICU setting. Generally, attempts at invasive treatment are not recommended at this time. The severe form of AP, including the presence of ACS symptoms, may continue for the entire duration of the disease, and interventional treatment may also be required in these patients.

From about the second week of AP, the severe form of the disease is usually associated with the development of various forms of pancreatic necrosis. In principle, a watch‑and‑wait approach is recommended in the cases of noninfected necrosis, provided no symptoms of obstruction, compression, or other complications, are present. It should be borne in mind that necrosis can spontaneously resorb and heal. The clinical presentation changes dramatically when local infection develops, as such a condition may require appropriate intervention to be considered on a case‑by‑case basis. In all patients with pancreatic necrosis, intervention is usually considered not earlier than at 4 weeks after the onset of the disease. It is recommended to defer intervention as long as possible, preferably until the signs of necrosis infection develop and the process comes to a natural containment with the subsequent demarcation of necrotic tissue, as this greatly facilitates their procedural removal. This is because the implemented surgical treatment should aim to remove all the fully differentiated necrotic foci and fluid reservoirs.

The principles of intervention listed below should be followed in the cases of severe AP.

- Normally, due to the time required for limited, demarcated necrosis to form, invasive treatment should be planned and implemented not earlier than in the fourth week after the onset of disease symptoms. If the necrotic tissues are not sufficiently demarcated, surgery should be avoided, as it may cause significant damage to adjacent tissues and a significantly greater loss of blood.
- If infected necrosis is diagnosed or even clinically suspected, it is advisable to consider surgical treatment in the patients whose clinical condition or general symptoms resulting from pro‑ longed MODS worsen despite optimum extensive conservative treatment.
- In patients with infected fluid reservoirs, pancreatic necrosis, and persistent MODS symptoms, if extensive conservative treatment is not effective, invasive rescue management is admissible prior to the fourth week of illness. A retrospective study by Zhang et al(Zhang et al., 2022) showed that invasive management within the first few weeks does not significantly worsen the final outcomes in patients with necrotizing forms of AP. In such cases, the aim of drainage is to evacuate infected contents to potentially reduce the risk of sepsis and help resolve other symptoms (such as pain or obstruction).
- In the cases of MODS not resolving despite optimum extensive treatment administered over several weeks after the onset of AP and in the absence of confirmed infection of pancreatic necrosis, surgery is allowed (optimum intervention when the necrosis is walled‑off and demarcated).
- Intervention is allowed at any stage of the disease if symptoms resulting from the mass effect of the necrotic focusoccur, including a sterile necrotic focus (pain, symptomatic obstruction of the stomach, intestines or biliary tract).
- Urgent or immediate intervention, regardless of the duration of the disease, is required in patients diagnosed with acute complications of AP, such as:
  - massive intra‑abdominal hemorrhage;
  - gastrointestinal tract perforation;
  - abscesses within the abdominal cavity;
  - mechanical bowel obstruction;
  - gastrointestinal hemorrhage due to venous thrombosis or other causes;
  - pseudoaneurysm of the splenic or gastro‑ duodenal artery or other visceral arteries (in these cases, the mortality rate is 34%–52%);
  - intestinal ischemia;
  - ACS not amenable to conservative treatment, with blood pressure >25 mm Hg or with concomitant MODS.

In conclusion, it should be noted that the literature lacks any conclusive data from RCTs or meta‑analyses documenting the advantage of the deferral of surgical intervention for infected pancreatic necrosis. The timing of the first intervention in the course of necrotizing AP remains a controversial issue. Both data suggesting maximum deference of intervention until demarcation of necrotic foci (>4 weeks) following appropriate antibiotic therapy (step‑up approach) and data indicative of the benefit of interventions applied shortly after the diagnosis of necrotic infection are available.(Boxhoorn et al., 2020)^,^(Tenner et al., 2024)^,^(Song & Lee, 2024)^,^(Sagar et al., 2022)^,^(Heckler et al., 2021)^,^(Yang et al., 2023)^,^(Nakai et al., 2022)

## 18. STEP‑UP APPROACH

### 18.1. We suggest that the interventional treatment of local complications of AP be started with percutaneous or endoscopic drainage techniques followed by subsequent re‑evaluation of indications for further, more invasive surgical treatment. (GRADE 1B)

The essence of the step‑up strategy for the surgical treatment of AP complications consists in an approach involving the implementation of surgical intervention when conservative treatment (usually antibiotic therapy, in this case referred to as step 0) has ceased to provide therapeutic benefit. The interventional regimen itself (step 1) begins with the least traumatic, endoscopic or percutaneous drainage methods, which can be repeated on an ad hoc basis if they offer a chance of a positive therapeutic effect. A systematic review of 11 credible studies analyzing the results of percutaneous drainage of the peripancreatic space showed satisfactory therapeutic effects in as many as 51% of patients, allowing the planned surgical intervention to be abandoned or postponed until the demarcation phase was reached, that is, when the risk of complications associated with the surgery itself was reduced.(Baron et al., 2020) On the other hand, in the absence of expected clinical improvement following drainage, it is recommended that qualification for another, more invasive intervention (laparoscopic; step 2) or, if unsuccessful, classic intervention through laparotomy (step 3) be considered. All of the above‑ mentioned procedures should be performed only if surgical treatment is indicated.

### 18.2. We propose that minimally invasive techniques be used in clinical cases requiring the removal of infected necrotic tissue. (GRADE 1B)

Minimally invasive methods also be beneficial in the treatment of AP complications, as they are associated with reduced surgical trauma. Step‑up surgical strategies, such as endoscopic transgastric necrosectomy or videoscopy‑assisted extraperitoneal necrosectomy (video‑assisted retroperitoneal debridement [VARD]), translate into reduced postoperative inflammatory response and a reduction in organ failure, as compared with open methods, while possibly involving a greater number of interventions.(Gurusamy et al., 2016)

## 19. STEP‑UP APPROACH: ENDOSCOPIC TREATMENT OF COMPLICATIONS

When qualifying patients for endotherapy of postinflammatory pancreatic and peripancreatic fluid reservoirs, we recommend that the endoscopic guidelines by Jagielski M et al (Guidelines for endoscopic treatment of post‑inflammatory pancreatic and peripancreatic fluid collections; in preparation) be followed.

### 19.1. When qualifying patients for endoscopic treatment of AP sequelae in the form of postinflammatory pancreatic and peripancreatic fluid reservoirs, we recommend that establishing an appropriate drainage system be the cornerstone of therapy. (GRADE 2B)

### 19.2. We recommend that the choice of drainage method be dependent on the location and type of postinflammatory pancreatic or peripancreatic fluid reservoirs. (GRADE 2B)

### 19.3. We propose endoscopic transmural drainage (through the wall of the upper gastrointestinal tract— transgastric or transduodenal) under EUS guidance as the preferred endotherapeutic approach. (GRADE 2B)

### 19.4. We recommend that active drainage through a nasal drain be used in the case of fluid reservoirs in the course of necrotizing AP. (GRADE 2B)

### 19.5. We propose that a step‑up approach be followed in the case of fluid reservoirs in the course of necrotizing AP, including potential increase in the number of the points of access to the necrotic tissue or intensification of treatment by means of direct endoscopic necrosectomy. (GRADE 2B)

Being one of the minimally invasive techniques for the management of fluid reservoirs resulting from pancreatitis, endoscopic treatment facilitates access to the reservoir via a transmural (through the gastric or duodenal wall) or trans‑ papillary (through the minor or major duodenal papilla) route. The mainstay of endoscopic treatment of postinflammatory pancreatic and peri‑ pancreatic fluid reservoirs is transmural drainage performed under EUS guidance through the up‑ per gastrointestinal wall.

For walled‑off pancreatic necrosis, infected PPCs, or sterile PPCs >10 cm in diameter, passive percutaneous drainage is insufficient, and active percutaneous drainage involving the insertion of a nasal drain into the reservoir cavity through percutaneous anastomosis for active flushing in the postoperative period was proven necessary. In some situations, intensification of endoscopic treatment in the form of endoscopic necrosectomy may be necessary. It involves inserting an endoscope through a transmural wall anastomosis into the lumen of the vessel under CO_2_ insufflation and removing dead tissue under endoscopic imaging guidance using various types of endoscopic instruments.(Costa DW et al., 2014)^,^(Arvanitakis et al., 2018)^,^(Onnekink et al., 2022)^,^(Baron et al., 2020)^,^(Jagielski et al., 2020)^,^(Gilman & Baron, 2023).

It should borne in mind that, according to the data available to date, the endoscopic step‑up approach has not proved more effective in long‑term follow‑up than the surgical step‑up approach as regards the reduction in rates of mortality or major complications in patients with infected necrosis in the course of AP. However, an overall lower number of pancreatic cutaneous fistulas was observed in endotherapy patients; they also required fewer re‑interventions over a longer follow‑up period. Several reports were also published that indicated the advantage of endoscopic management in a step‑up strategy, in which the role of laparoscopic technique (step 2) is taken over by endoscopy. Endoscopic drain‑ age is characterized by a lower rate of complications, including the development of fistulas, lower treatment costs, and greater patient comfort (PENGUIN and MISER studies).(Sobocki et al., 2023)^,^(Bang et al., 2019)^,^(Timmermann et al., 2024)^,^(Bang et al., 2020)

The above data may suggest that the minimally invasive treatment of patients with the sequel‑ ae of necrotizing pancreatic and peripancreatic fluid reservoirs should start with endoscopic techniques, and be modified toward percutaneous access and surgical techniques only if the former are proved ineffective.

## STEP‑UP APPROACH: SURGICAL TREATMENT OF COMPLICATIONS

The optimum invasive treatment method for suspected infected pancreatic necrosis remains undetermined.

In principle, the following surgical techniques are acceptable for treating AP complications (de‑ pending on the experience of the surgical team and local condition):

- percutaneous retroperitoneal or transperitoneal drainage;
- minimally invasive retroperitoneal necrosectomy;
- video‑assisted retroperitoneal debridement (retroperitoneal access—VARD, transgastric access);
- laparoscopic necrosectomy;
- open necrosectomy;
- laparoscopic c ystogastrostomy or cystojejunostomy;
- open cystogastrostomy or cystojejunostomy.

### 20.1. In clinical cases requiring removal of infected necrotic tissue, we suggest that the minimally invasive technique in which the particular institution has the greatest experience be the method of choice. (GRADE 1B)

The use of minimally invasive surgery techniques to treat infected pancreatic necrosis was declared a standard when the results of the PANTER RCT were published, demonstrating the superiority of minimally invasive interventions over open surgery in terms of early and distant outcomes.(Boxhoorn et al., 2020) As of present, the most common types of access facilitating drainage or removal of infected necrotic pancreatic parenchyma or peripancreatic tissues include: 1) percutaneous drainage; 2) endoscopic drainage; 3) videoscopic procedures (such as VARD or laparoscopic transgastric debridement). The primary minimally invasive surgical procedure is VARD, the efficacy of which was confirmed in the PANTER RCT.(Santvoort HC et al., 2010) Laparoscopic technique has been successfully used in the second stage of the step‑up ladder, after the failure of either percutaneous or endoscopic drainage. According to most reports with high strength of evidence, no data are available at this time to unequivocally identify a particular surgical intervention technique with the greatest therapeutic efficacy. It follows that the intensive treatment regimen should be individualized on a case‑by‑case basis, in principle consisting of a combination of various accesses and techniques. At the same time, given the perioperative mortality rate in severe forms of AP, insufficient data are also available to support a confident recommendation for a particular type of primary surgical intervention (ie, open, minimally invasive, or endoscopic).

Based on the available publications, the superiority of the surgical step‑up algorithm over the endoscopic one cannot be conclusively demonstrated with regard to severe complications or mortality; however, given the results of the EXTENSION RCT, it appears that endoscopic procedures are associated with an overall lower number of complications in the form of pancreatic cutaneous fistulas as well as fewer reinterventions after 6 months of follow‑up.(Onnekink et al., 2022) The only RCT comparing the step‑up therapeutic approach with traditional open necrosectomy demonstrated the superiority of the minimally invasive regimen in long‑term follow‑up. In the study groups, 44% of patients died or experienced serious complications after the step‑up strategy, as compared to as many as 73% of patients dying after traditional surgery in the control group (P = 0.005). A significantly lower percentage of patients in the step‑up treatment arm developed postoperative hernias (23% vs 53%; P = 0.004), exocrine pancreatic insufficiency (29% vs 56%; P = 0.03), and endocrine failure (40% vs 64%; P = 0.05). At the same time, no significant differences were observed between the groups in terms of the rates of additional drainage procedures (11% vs 13%; P = 0.99) or pancreatic surgeries required (11% vs 5%; P = 0.43); similarly, no differences were observed in terms of recurrent AP, chronic pancreatitis, pain levels, or treatment costs. In both groups, quality of life improved subjectively during the follow‑up period while not reaching the level of statistical significance.(Hollemans et al., 2019)

### 20.2. We do not recommend routine use of open abdominal surgery in patients with AP without concomitant ACS. (GRADE 2C)

Traditional laparotomy is currently recommended only for acute complications, such as gastrointestinal tract perforation, intestinal ischemia, mechanical obstruction, or intra‑abdominal hemorrhage. In such cases, the type of surgical access (medial vs transverse incision) is dictated by the experience of the center and the current clinical condition of the patient.

Another indication for laparotomy being pursued in surgical treatment for AP complications is the failure of less invasive treatment modalities as part of the step‑up strategy.

If conservative treatment of ACS proves ineffective, decompression procedures are also recommended; in the case of failure, laparotomy surgery involving negative‑pressure wound healing techniques is recommended.

The choice of the surgical treatment of AP complications, including the open abdominal approach, flow drainage, and elective relaparotomies, shall always be determined by the experience of the center and the clinical setting.

### 20.3. We recommend that negative pressure dressings be used in patients treated with open abdominal surgery. (GRADE 2C)

No high‑quality evidence is available to support the use of negative pressure for open abdominal treatment in AP patients. Isolated observational reports are available on the benefits of such therapy.(Wen et al., 2023)

## 21. CRITERIA FOR COMPLETION OF INPATIENT TREATMENT

### 21.1. We propose that the inpatient treatment of patients with severe AP be completed in accordance with the Hungarian Pancreatic Study Group criteria after the completion of invasive treatment and antibiotic therapy and with patient’s overall condition being taken into account. (GRADE 2C)

Insufficient scientific data are available in the literature to establish the criteria for hospital discharge of patients treated for severe AP.

In 2023, the Hungarian Pancreatic Study Group reported on a multicenter international survey and cohort study focused on the hospital discharge protocol of patients with mild AP. The protocol was based on clinical evaluation and laboratory test results (CRP levels and pancreatic enzyme activities). According to the protocol, preparation for the completion of hospitalization can be pursued in patients whose abdominal pain has been alleviated, CRP and pancreatic enzyme levels have been reduced, while no contra indications to oral nutrition exist. If no pain complaints are observed within 24 hours after the implementation of nutrition and the pancreatic enzyme activity and CRP levels are reduced to <50 mg/dl, the patient can be discharged from the hospital, provided no other contraindications, such as the need for continued antibiotic therapy or endoscopic procedures, exist. If CRP levels persist at >50 mg/dl, the patient’s condition should be re‑evaluated after 24 hours. If no pain is observed and the pancreatic enzyme activity and CRP levels are further reduced, the patient can be discharged home even if the latter has not fallen below <50 mg/dl.(Nagy et al., 2023)

## 22. PROGNOSIS AND OUTPATIENT SURVEILLANCE

Due to the risk of recurrent AP, the increased risk of exo- and endocrine failure and of the diagnosis of pancreatic cancer and fluid reservoirs, patient follow‑up should be continued even after the completion of the primary AP treatment. An additional benefit of such surveillance consists in the possibility of earlier detection of pancreatic cancer as the cause of the disease.

### 22.1. We recommend that patients be followed for up to 2 years after the completion of AP treatment. (GRADE 1C)

In a prospective cohort study of 1184 individuals having undergone AP treatment, de Rijk et al212 observed inflammation recurring in 25% of those observed, with the median time to recurrence amounting to 9 months. Chronic pancreatitis, on the other hand, was detected in 6% of patients, with the median time to the diagnosis in this group amounting to 31 months. Diagnosis of pancreatic cancer was established in 1% of patients, on average after about 24 months of follow‑up.

### 22.2. We recommend that follow‑up abdominal US be performed at 3 and 6 months after hospital discharge. (GRADE 1C)

A history of AP is associated with an increased risk of local fluid reservoir and PPC formation. Peri‑ pancreatic fluid reservoirs are found in as many as 34%–43% of patients, while PPCs are found in 6%–10%, with spontaneous resorption observed in about 70% of cases. The prognostic factors for the formation of fluid reservoirs and PPCs include alcohol‑related background of AP, a severe course of the disease, and high CRP levels at 48 hours after admission to the hospital. The determinants of spontaneous cyst regression include a non symptomatic or oligosymptomatic course of post‑ hospitalization follow‑up and the cyst diameter of <4 cm. A prospective study showed that flu‑ id reservoirs were present in 34% of patients dis‑ charged from the hospital while PPCs were visualized on follow‑up US scans acquired 3 and 6 months after discharge in 10% and 7% of patients, respectively.(Lankisch et al., 2012)^,^(Cui et al., 2014)

### 22.3. In the cases of BISAP score of ≥2 and CTSI (Balthazar) score of ≥3, we recommend that contrast‑enhanced CT scan be acquired at 1–3 months after hospital discharge. (GRADE 1C)

Based on a 2015 study, Cho et al(Cho et al., 2015) concluded that due to the possibility of detecting fluid reservoirs and pancreatic cancer as a cause of AP, early follow‑up involving CT and possibly US imaging is warranted in some AP survivors. A CT scan should be performed 1–3 months after the patient is discharged from the hospital. Factors indicative of the particular appropriateness of such treatment include the BISAP score of ≥2 and CTSI (Balthazar) score of ≥3.(Cho et al., 2015)

### 22.4. Due to the increased risk of pancreatic cancer, we suggest that CT scans be repeated after 1 and 2 years. (GRADE 1C)

In a population‑based study of South Korean patients, Park et al(Park et al., 2023) observed a significantly higher risk of pancreatic cancer in a group of AP survivors, as compared with a control group: 1.9% vs 0.2%. The risk was 8.46 times higher at 1–2 years after the onset of AP and 3.62 times higher at 2–4 years after the onset of AP. The differences in the pancreatic cancer risk disappeared only after 10 years since the episode of AP.(Park et al., 2023)

### 22.5. If exocrine pancreatic insufficiency is suspected, we recommend that the activity of fecal elastase 1 (FE‑1) be assessed by means of an FE‑1 assay or, alternatively, a 13C‑labeled mixed triglyceride breath test. (GRADE 1C)

Exocrine pancreatic insufficiency is found in 19.4% of patients after mild AP and 33.4% of patients after severe AP. It is more common in patients with biliary AP of alcoholic etiology than in patients with biliary AP or AP of other etiologies: 22.7% vs 10.2% vs 13.4%, respectively. Exocrine pancreatic insufficiency is mainly diagnosed on the basis of the fecal elastase 1 (FE‑1) test or, alternatively, a 13C‑labeled mixed triglyceride breath test.(Hollemans et al., 2018)^,^(Domínguez‐Muñoz et al., 2017)^,^(Domínguez‐Muñoz et al., 2016)

### 22.6. We recommend monitoring of endocrine pancreatic insufficiency as manifested by prediabetes or diabetes (type 3c) for 1–2 years after the episode of AP on the basis of fasting blood glucose, glycated hemoglobin (HbA1c), and glucose load test. (GRADE 1C)

Endocrine pancreatic failure as manifested by pre‑ diabetes or diabetes (type 3c) develops in 37% of patients after the first episode of AP. The risk of prediabetes is 16%, and the risk of diabetes is 23%. About 15% of these patients require insulin treatment. Most frequently (in 15% of patients), diabetes is diagnosed up to 12 months after the onset of AP, while an increased risk of impaired glucose tolerance persists for up to 5 years after the episode. The prediabetes and diabetes screening pro‑ tocol should be based on fasting blood glucose and HbA1c monitoring and the glucose load test.(Das et al., 2014)^,^(Ewald & Bretzel, 2013)

## 23. SUMMARY

Severe AP is a disease characterized by multifactorial etiology and pathogenesis, and a very dynamic clinical course requiring a multidisciplinary diagnostic approach, close monitoring, and treatment tailored to the clinical situation. An extremely important element in the planning of the therapeutic pathway consists in determining the timing of interventional treatment, in which a step‑up approach should be followed. Severe forms of AP should be treated at centers having the experience and capacity to provide patients with appropriate care in accordance with current management principles.
